# Versatile microbial communities rapidly assimilate ammonium hydroxide-treated plastic waste

**DOI:** 10.1093/jimb/kuad008

**Published:** 2023-04-14

**Authors:** Laura G Schaerer, Emily Wood, Sulihat Aloba, Emily Byrne, M Aamir Bashir, Kaushik Baruah, Elizabeth Schumann, Libby Umlor, Ruochen Wu, Hyeonseok Lee, Christopher J Orme, Aaron D Wilson, Jeffrey A Lacey, Rebecca G Ong, Stephen M Techtmann

**Affiliations:** Department of Biological Sciences, Michigan Technological University, Houghton, MI 49931, USA; Department of Biological Sciences, Michigan Technological University, Houghton, MI 49931, USA; Department of Chemical Engineering, Michigan Technological University, Houghton, MI 49931, USA; Department of Biological Sciences, Michigan Technological University, Houghton, MI 49931, USA; Department of Chemical Engineering, Michigan Technological University, Houghton, MI 49931, USA; Department of Chemical Engineering, Michigan Technological University, Houghton, MI 49931, USA; Department of Chemical Engineering, Michigan Technological University, Houghton, MI 49931, USA; Department of Chemical Engineering, Michigan Technological University, Houghton, MI 49931, USA; Department of Chemical Engineering, Michigan Technological University, Houghton, MI 49931, USA; Biological Processing Department, Idaho National Laboratory, Idaho Falls, ID 83415, USA; Biological Processing Department, Idaho National Laboratory, Idaho Falls, ID 83415, USA; Biological Processing Department, Idaho National Laboratory, Idaho Falls, ID 83415, USA; Biological Processing Department, Idaho National Laboratory, Idaho Falls, ID 83415, USA; Department of Chemical Engineering, Michigan Technological University, Houghton, MI 49931, USA; Department of Biological Sciences, Michigan Technological University, Houghton, MI 49931, USA

**Keywords:** Plastic, Biodegradation, Microbial community, Bioprocessing, Upcycling

## Abstract

Waste plastic presently accumulates in landfills or the environment. While natural microbial metabolisms can degrade plastic polymers, biodegradation of plastic is very slow. This study demonstrates that chemical deconstruction of polyethylene terephthalate (PET) with ammonium hydroxide can replace the rate limiting step (depolymerization) and by producing plastic-derived terephthalic acid and terephthalic acid monoamide. The deconstructed PET (DCPET) is neutralized with phosphoric acid prior to bioprocessing, resulting in a product containing biologically accessible nitrogen and phosphorus from the process reactants. Three microbial consortia obtained from compost and sediment degraded DCPET in ultrapure water and scavenged river water without addition of nutrients. No statistically significant difference was observed in growth rate compared to communities grown on DCPET in minimal culture medium. The consortia were dominated by *Rhodococcus* spp., *Hydrogenophaga* spp., and many lower abundance genera. All taxa were related to species known to degrade aromatic compounds. Microbial consortia are known to confer flexibility in processing diverse substrates. To highlight this, we also demonstrate that two microbial consortia can grow on similarly deconstructed polyesters, polyamides, and polyurethanes in water instead of medium. Our findings suggest that microbial communities may enable flexible bioprocessing of mixed plastic wastes when coupled with chemical deconstruction.

## Graphical Abstract

**Graphical Abstract:**
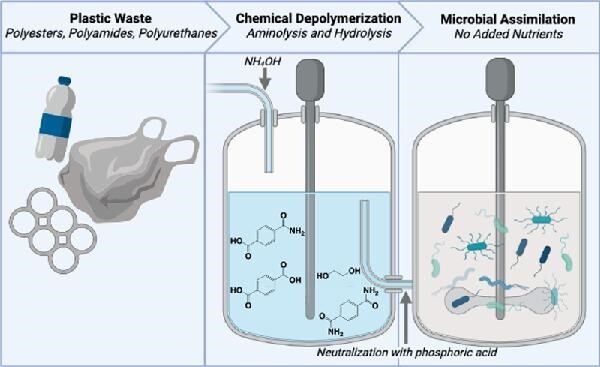
Natural microbial communities are able to flexibly convert deconstructed plastic wastes into biomass.

## Significance

Plastic waste has been shown to be a valuable feedstock for coupled tandem chemical and biological processing. Often, these processes require the use of a defined bacterial culture medium and are optimized for a single plastic type using a single bacterial strain. Here, we demonstrate the ability of microbial consortia to metabolize chemically deconstructed PET. Furthermore, we show that by strategically choosing chemical reagents for chemical pre-processing of polyethylene terephthalate, we can streamline the coupled chemical deconstruction and microbial biodegradation system to process plastic waste for upcycling or microbial assimilation. This process is flexible and can work for diverse plastic types using a single microbial consortium. Results presented here suggest that the flexibility of a microbial community may allow for simultaneous processing of mixed plastic waste. This study can inform future work on microbial upcycling of waste polymers into more valuable products and bioremediation of waste streams.

## Introduction

Each year, over 350 million tons of plastic are produced globally (Geyer et al., 2017; Jambeck et al., 2015). Over 70% of plastic waste is disposed of in landfills or accumulates in natural environments, particularly the ocean (Geyer et al., 2017; Jambeck et al., 2015). This massive growth in plastic waste calls for improved methods for recycling of plastic, as many of the current methods for recycling lead to downcycling of the plastic (the production of a product that is of lower quality or functionality than the original material) (Garcia & Robertson, 2017; Jambeck et al., 2015). Polyethylene terephthalate (PET) is one of the most common plastics worldwide due to its low cost and durability. This plastic polymer consists of repeating terephthalate and ethylene glycol units connected through ester linkages (Jeyakumar et al., 2013). PET is widely used for both food packaging and polyester blend fabrics (Parab & Shukla, 2013). Over 41 million metric tons of PET were produced globally in 2014, with future production of PET expected to grow exponentially (PET Global Production Forecast 2020, n.d.). There has been a great deal of recent progress toward engineering PET-hydrolyzing enzymes to depolymerize PET waste for reprocessing into virgin PET (Arai et al., 2010; Chamas et al., 2020; Müller et al., 2001). However, enzymatic processes generally require the PET to be a thin film (Chamas et al., 2020) or ground into a powder to enable access of the enzyme to the plastics (Arai et al., 2010; Chamas et al., 2020), which adds to energy consumption and processing costs. Despite advances toward a circular plastic economy, additional approaches for PET recycling and degradation are needed to supplement these advances. As current recycling technologies are optimized and additional technologies are developed and adopted that degrade PET, it may be possible that PET pollution of natural environments will decrease and energy resources will be preserved (Geyer et al., 2017).

While techniques currently exist for chemical and biological recycling of PET, current chemical recycling techniques require the addition of catalysts and often use extreme processing conditions to depolymerize and reprocess PET into virgin material or other value-added products (Schaerer et al., 2022; Singh et al., 2021). Likewise, biological processing requires the addition of defined nutrient-rich media to support microbial growth. The addition of chemical catalysts for pre-processing, as well as the addition of culture medium to support microbial growth, adds considerable expense to the overall processing of PET waste (Singh et al., 2021).

Previous work has shown the promise of coupling chemical and biological processing of plastic waste to upcycle plastic waste into value-added products (Byrne et al., 2022; Sullivan et al., 2022). PET can be depolymerized via several different chemical treatments using strong alkali, such as sodium hydroxide (Geyer et al., 2016; Karayannidis & Achilias, 2007; Khalaf & Hasan, 2012; Paliwal & Mungray, 2013). However, the strong alkaline conditions required are highly corrosive and require specialized materials for process equipment and extra maintenance, which can be expensive (Zenda & Funazukuri, 2008). Ammonium can also be used to deconstruct the PET through aminolysis into terephthalamide and ethylene glycol (Shukla & Harad, 2006). When ammonium hydroxide is used, if the reaction proceeds to completion, a mixture of monomers is formed due to competing aminolysis and hydrolysis reactions, yielding a final product consisting of terephthalamide, terephthalic acid, terephthalic acid monoamide, and ethylene glycol (Paliwal & Mungray, 2013; Shukla & Harad, 2006). Researchers have reported increased deconstruction rates of plastic particles in a dilute aqueous ammonium solution compared to hydrolysis using sodium hydroxide (Arai et al., 2010). By strategically using ammonium hydroxide to depolymerize PET, it may be possible to produce nitrogen-rich biodegradable plastic derivatives for microbial community degradation. Additionally, the alkaline product from chemical depolymerization could be neutralized with phosphoric acid, yielding a pH-neutral feedstock rich in nitrogen and phosphorus (Schaerer et al., 2022).

Biological degradation of PET is possible with or without chemical pretreatment, although chemical pretreatment increases biodegradation rates. Enzymatic degradation of PET has been observed by both naturally-occurring and engineered microorganisms (Geyer et al., 2017; Jambeck et al., 2015). In natural environments, the decomposition of PET and other aromatic compounds is very slow (Chamas et al., 2020; Müller et al., 2001). The model PET degrader *Ideonella sakaiensis* can depolymerize and degrade PET (Fecker et al., 2018; Son et al., 2019); however, this process is inefficient even under laboratory conditions. For example, *I. sakaiensis* can almost completely degrade PET films after 6 weeks of incubation at 30 °C (Yoshida et al., 2016), which is prohibitively slow for the purpose of industrial-scale plastic biodegradation. Previous research has suggested that it is unlikely for naturally occurring microorganisms alone to degrade PET on an industrial scale (Wright et al., 2021). In contrast to growth on polymers, growth on depolymerized PET derivatives, such as terephthalate, is much more efficient. For example, a mixed culture of *Pseudomonas* sp. C4S and *Bacillus* sp. C4B showed complete degradation of terephthalate after only 19 hr of incubation at 50 °C (Roberts et al., 2020). Terephthalate-degrading organisms are widespread and have been isolated from numerous sources, including soil (Suwanawat et al., 2019), household compost (Sulaiman et al., 2012), and wastewater (Kimura & Ito, 2001). Examples of terephthalate-degrading isolates include *Alcanivorax* (Delacuvellerie et al., 2019), *Syntrophus* (Morris et al., 2013) genera within the Proteobacteria; *Pelotomaculum* (Morris et al., 2013) genus within the Firmicutes; and *Rhodococcus* (Suwanawat et al., 2019) genus within the Actinobacteria. Chemical depolymerization of PET offers a faster alternative to naturally occurring PETases by chemically deconstructing PET polymers into smaller molecules, which are more easily biodegradable (Müller et al., 2001; Paliwal & Mungray, 2013). Tandem processing takes advantage of the speed of chemical deconstruction to convert plastic waste into more biologically accessible monomers, ultimately increasing the rate of bioconversion while allowing PET to be upcycled into valuable chemicals (Byrne et al., 2022; Shah et al., 2008; Werner et al., 2021).

Here, we explore the potential to couple ammonium hydroxide treatment of PET with biological processing by microbial communities. While ammonium hydroxide treatment of the plastics could depolymerize the plastic and provide a nutrient source, there are still challenges that must be overcome. One potential challenge to the coupled chemical and biological processing of PET using aminolysis is that terephthalamide is one of the expected deconstruction products of PET. Terephthalamide has been shown previously to have antimicrobial properties (Watanabe, 1991), and very little is known about the biochemical pathways involved in the degradation of terephthalamide, or if terephthalamide is biodegradable (Watanabe, 1991). Another challenge of processing PET with ammonium hydroxide is the resultant mixture of compounds. This mixture would need to be degraded by either an organism or microbial community capable of degrading all of the expected deconstruction products.

Often metabolism of complex substrate mixtures will require cooperation within a microbial community (Morris et al., 2013; Yu et al., 2019). Microbial communities have been used for industrial purposes for decades, for example: treating wastewater (American Public Health Association et al., 1999), producing renewable energy (Lau & Dale, 2009), and manufacturing fermented dairy products. Division of labor in microbial communities allows communities to be flexible and limits metabolic burden on any single organism (Qi et al., 2021). Research has shown that community interactions can improve the rates of plastic biodegradation, suggesting that a microbial community will be able to degrade plastic more efficiently than a single isolate working alone (Meyer-Cifuentes et al., 2020; Roberts et al., 2020; Wanapaisan et al., 2018). However, much of the previous work related to upcycling of PET is focused on isolates due to the ease of genetically engineering single organisms to convert PET into value-added compounds (Sadler & Wallace, 2021). Many methods for upcycling waste using microorganisms have been previously considered: pyrolysis-aided microbial community conversion of plastic into food (Byrne et al., 2022), conversion of agricultural waste into fuel using *Saccharomyces cerevisiae* (Lau & Dale, 2009), or using enzymes to convert waste PET back into virgin PET (Lu et al., 2022). It is possible that the mixture of products resulting from aminolysis and hydrolysis of PET may require more than one species of microorganism for complete and efficient metabolism. Recent studies have shown that tailoring the chemical deconstruction process to produce specific chemicals can be used in tandem chemical and bioprocessing (Guzik et al., 2014; Sullivan et al., 2022; Werner et al., 2021). One advantage of microbial communities is their flexibility to degrade complex substrates.

The primary goal of this paper is to determine the potential for microbial communities to be used to process chemically pre-treated PET waste. Here, we seek to explore how a microbial community may be able to degrade more than one type of chemically deconstructed plastic, allowing for the processing of mixed plastic waste in a single system. Because the process for deconstructing PET demonstrated here results in a product rich in nitrogen and phosphorus, we expect the solution will be biodegraded without specialized medium or additional nutrients. We hope this streamlined process will form the basis of future industrial systems to upcycle PET waste into valuable products (such as food) before it pollutes the environment (Schaerer et al., 2022). Additionally, ammonium hydroxide treatment may also be used to deconstruct additional polymers, such as flexible polyurethane foam fragments, polycarbonate shards, polyester layers in multilayer plastics, and synthetic fabrics (pure nylon or polyester and nylon- and polyester-spandex blends). We hypothesize that (1) our naturally enriched microbial consortium will be able to grow on and degrade the mixture of plastic derivatives in the deconstructed PET (DCPET), (2) the nutrient-rich DCPET will support microbial growth without additional nutrients, and (3) the microbial consortium will grow on additional ammonium hydroxide-treated polymers.

## Materials and Methods

### Brief Summary of Methods

PET plastic was chemically deconstructed using ammonium hydroxide. The products of the DCPET were quantified using high-performance liquid chromatography (HPLC). Microbial consortia were enriched to degrade the model compound, terephthalate. Three terephthalate-degrading consortia (LS1_Calumet, LS2_Calumet, and EB2_Mackinac) were used to test whether products of PET deconstructed with aminolysis and hydrolysis were biodegradable. For each of the three cultures, four culture conditions were set up in triplicate: (1) Bushnell–Haas (BH) medium amended with the model compound terephthalate, (2) BH medium amended with DCPET, (3) sterile ultra-pure water amended with DCPET, and (4) sterilized river water amended with DCPET. Nutrients (ammonium, nitrate, nitrite, soluble reactive phosphorus, total dissolved nitrogen, and total organic carbon) were measured for each of the four media types prior to inoculation. During growth, daily subsamples were collected to measure OD_600_. After 7 days of growth, the cultures were sacrificed for additional analysis: (1) DNA extraction and subsequent 16S rRNA sequencing, and (2) HPLC quantification of remaining substrates).

Preliminary data was also collected to test whether this process may also work for similar polymers, specifically polyurethane foam, polycarbonate, polyester, nylon, and spandex. Additional polymers were deconstructed using ammonium hydroxide, and the products were used to perform growth tests in sterile water with two microbial consortia (LS1_Calumet and EB9_Mackinac).

### Generation of Consortia

All microbial consortia were enriched to degrade terephthalate from compost and sediment samples. The LS1_Calumet and LS2_Calumet replicate cultures were obtained from compost collected from a farm in Calumet, MI (coordinates 47.211, −88.553). Initially, cultures were started by adding 1 g of compost per 100 mL of 10 g/L disodium terephthalate in BH medium containing the following: magnesium sulfate (0.2 g/L), anhydrous calcium chloride (0.02 g/L), potassium dihydrogen phosphate (1 g/L), dipotassium hydrogen phosphate (1 g/L), ammonium nitrate (1 g/L), and ferric chloride (0.05 g/L). Cultures were incubated at room temperature, stirred continuously with Teflon-coated magnetic stir bars at 130 rpm. Every 14 days, enrichments LS1_Calumet and LS2_Calumet were used to inoculate fresh 10 g/L disodium terephthalate in BH medium (10% inoculum) for a total of four transfers. After the enrichment process was complete, enrichments were maintained in 1 L volumes, and 400 mL of spent culture was replenished with 400 mL of fresh 10 g/L disodium terephthalate in BH medium every 3–7 days. The EB2_Mackinac culture was enriched from sediment collected from the straits of Mackinac (coordinates 46.532, −88.141) and the same compost sample as the LS1_Calumet and LS2_Calumet enrichments. The culture was started by adding 125 µL of pyrolysis-treated polypropylene products [previously described by Byrne et al. (2022)] to BH media with 2 g of each inoculum. The culture was incubated at room temperature and stirred continuously at 200 rpm. The culture was transferred daily for 5 days, and then maintained in the same manner as the LS1_Calumet and LS2_Calumet cultures. EB9_Mackinac is a lower diversity community that was derived from the EB2_Mackinac community used for earlier experiments. The community is further described in the supplemental materials (Supplementary Fig. 10).

### Chemical Deconstruction of PET Using NH_4_OH

DCPET was produced from dimethyl ether-treated PET (diameter between 100 microns to 2 mm) was supplied by the Idaho National Laboratory. The full methods are described in the supplemental material. Aqueous ammonium hydroxide (NH₄OH: 28–30 wt%) was purchased from Sigma-Aldrich and mixed with distilled water to the appropriate concentration. Raw PET was loaded with 10 wt% NH_4_OH at 0.25 g PET/mL NH_4_OH to 13.5 mL working volume in a custom 20 mL vertical batch reactor (Supplementary Fig. 8). Heating tape (HTS/Amptek) was wrapped outside the reactor to heat it up, and K-type thermocouples were used to measure the liquid temperature in the reactor. After loading the liquid, the reactor was heated to 240 °C and held for 60 min residence time before cooling the outside of the reactor using compressed air. The liquid product was filtered with Whatman #42 filter paper (diameter 55 mm, pore size 2.5 µm). Solid products remaining on the filter paper were dried at 55 °C in a Thermo Scientific 180 L gravity oven overnight. Liquid products were collected for analysis and then neutralized with the addition of phosphoric acid from a pH of 9.5 to 7. Upon neutralization, the DCPET formed a slurry and was centrifuged at 10 000× *g* for 30 min. The supernatant was decanted, and then the precipitate was centrifuged again at 10,000× *g* for 30 min to separate any additional liquid from the solid precipitate. The supernatant from both centrifugation steps was combined and used as the DCPET feedstock for cultures.

### Testing Growth of Enriched Consortia in Media, Sterile Water, and Sterile River Water

To test whether the microbial consortia could grow on DCPET derivatives under a variety of conditions, sterilized river water, BH medium, and ultrapure MilliQ water were obtained. River water was collected from the Pilgrim River in Chassell, MI, in June 2021 (coordinates 47.106, –88.513). The river water was sterilized by autoclaving and filtering through a 0.2-micron PES filter. BH medium was prepared according to the manufacturer's instructions.

For each of the three enrichments, three flasks were set up with triplicates for each of the three media types: autoclaved river water (ARW), autoclaved Milli-Q water (AMW), and BH media. These flasks were amended with DCPET. To achieve an estimated 10 g/L concentration of DCPET in 50 mL flasks, 2.5 mL of 0.2 g/mL DCPET was added to each of the DCPET treatments. The remaining three flasks in each group did not have any added carbon source. Additionally, for each enrichment, triplicate positive controls were prepared with 10 g/L disodium terephthalate in BH. Flasks were inoculated with a 10% inoculum (5 mL). Uninoculated flasks were also prepared as blanks. The final volume in each flask totaled 50 mL. The experimental design is shown in Fig. 6.

Flasks were incubated at 37 °C and stirred at 130 rpm with Teflon-coated magnetic stir bars. Daily, 200 μL samples were collected to measure OD_600_ to approximate growth. At the end of the experiment, 10 mL aliquots of cell culture were centrifuged at 10 000× *g* for 20 min, and the supernatant was decanted. The cell pellet was frozen at –20 °C until DNA extraction.

### Statistical Analysis of Growth Curves

To test whether there was a statistically difference in growth rate between treatments for each consortium, statistical analysis of growth curves was performed. Analysis of OD_600_ values was performed in R using tidyverse (Wickham et al., 2019) and base R (R Core Team, 2013). To test if the growth curves were statistically different between the DCPET treatments, we followed a tutorial from “R in Ecology and Evolution” (Campitor, 2011). An analysis of covariance (ANCOVA) was performed to evaluate if there was a statistically significant difference between the OD_600_ values, time, and treatment group. To ensure linear growth, only the first five OD_600_ time points were used for this analysis. Two ANCOVA tests were performed, one with an interaction term between time and treatment group and the other test with no interaction term, where the relationship between OD_600_ values and time was evaluated independently from the relationship between OD_600_ values and treatment group. Using an Analysis of Variance (ANOVA), the two models were compared to see if the interaction term added important information to the models. If the two models were statistically different, then the interaction term added important information to the model. For significant comparisons, a pairwise *t*-test with Bonferroni adjustment of the *p*-values was used as a post hoc test (Dunn, 1964). The code for this analysis can be found at https://github.com/lgschaer/full_CDPET.

### HPLC Analysis

To quantify the composition of the DCPET material before and after biodegradation, HPLC was performed. Terephthalic acid and terephthalic acid monoamide were purchased from Millipore-Sigma. Terephthalamide was purchased from TCI Chemicals. Bis(2-hydroxyethyl) terephthalate (BHET) was purchased from Sigma-Aldrich. The 4-((2-Hydroxyethoxy)carbonyl)benzoic acid [aka mono(2-hydroxyethyl) terephthalate (MHET)] was purchased from Advanced ChemBlocks. Standard solutions of terephthalic acid, terephthalic acid monoamide, terephthalamide, BHET, and MHET were prepared in N, N-dimethylformamide (DMF) at a concentration of 1 mg/mL. Terephthalic acid, terephthalic acid monoamide, BHET, and MHET were analyzed using the DAD at 300 nm, while terephthalamide was analyzed at 275 nm due to their optimum linear standard curves observed at these wavelengths. The stock solutions were further diluted with DMF and filtered through a 0.22 μm polytetrafluoroethylene filter to give five different concentrations for calibration curves. The calibration curves were obtained by plotting the peak area of each standard to their known concentrations.

The analysis was performed with an Agilent 1200 liquid chromatography system equipped with a G1311A quaternary pump, G1322A degasser, G1329A autosampler, G1315B DAD detector, and 61316A temperature column controller. The separations were carried out using a Waters μBondapak C18 column (3.9 × 300 mm, 10 μm). The mobile phase consisted of 0.2% formic acid-water solution (A) and 0.1% formic acid-acetonitrile solution (B) using a gradient elution: 0–5 min, 20–30% B; 5–10 min, 30–40% B; 10–15 min, 40–50% B; 15–20 min, 50–60% B; 20–25 min, 60–70% B; 25–35 min, 70–90% B; and 35–45 min, 90–20% B. The analysis was carried out at a flow rate of 0.4 mL/min and an injection volume of 20 μL over 45 min.

### Nutrient Analysis

To quantify the nutrient content of each treatment prior to biodegradation, nutrient quantification was performed. Nutrient analyses were performed at the AQUatic Analysis (AQUA) Lab at the Great Lakes Research Center at Michigan Technological University (AQUA Lab—Method Summaries, n.d.). Initial concentrations of ammonium, nitrate, nitrite, soluble reactive phosphorus, total dissolved nitrogen, and total organic carbon were quantified in ultrapure water, Pilgrim River water, and BH media, with and without addition of DCPET. The inorganic nutrients in ultrapure water and sterilized river water were determined with no dilution. The BH medium was diluted 1:1 000 prior to nutrient analysis. The samples with DCPET were diluted 1:100 000 and 1:1 000 000 prior to nutrient analysis to ensure that the nutrient concentrations were in the range of detection. The concentrations of the undiluted solutions were calculated.

Concentrations of ammonium, nitrate, nitrite, and soluble reactive phosphorus were analyzed with a SEAL Analytical AQ2 Discrete Analyzer (87–89). Ammonium was reacted with hypochlorite, liberated from dichloroisocyanurate to form a chloramine. The alkaline salicylate and chloramine react to form indophenol blue dye. Since the color is proportional to the concentration of ammonium, it is photometrically measured at 660 nm to estimate ammonium concentration. Nitrate is reduced to nitrite in a cadmium coil. Nitrite and chemically reduced nitrate are reacted with alkaline salicylate in the presence of hypochlorite and sodium nitroprusside, yielding a magenta azo dye that is measured photometrically at 520 nm. A complex of antimony-phospho-molybdate is formed when acidic molybdate and antimony potassium tartrate react in the presence of orthophosphate. Ascorbic acid reduction of this complex yields phosphomolybdenum blue, which can be measured photometrically. The phosphorus concentration is proportional to the color, quantified by absorbance at 880 nm.

Total dissolved nitrogen and total organic carbon are analyzed using Shimadzu TOC-L_CPH_ analyzer with TNM-L (American Public Health Association et al., 1999; AQUA Lab—Method Summaries, n.d.). To estimate total dissolved nitrogen, samples are combusted at 720°C to form nitrogen monoxide. The carrier gas delivers the nitrogen monoxide to a chemiluminescence gas analyzer, where a measurable peak is generated. Total organic carbon is measured by acidifying the sample to convert inorganic carbon to CO_2_, then sparged to remove the CO_2_. The remaining carbon is the non-purgeable organic carbon sample, which is injected into a combustion tube that contains oxidation catalyst and is combusted at 680 °C to convert the organic carbon into CO_2_. Carbon-free carrier gas delivers the CO_2_ to a non-dispersive infrared gas analyzer, which measures peaks to estimate the amount of carbon.

### 16S rRNA Sequencing

To explore the community composition of each consortium in each treatment, 16S rRNA sequencing was performed. DNA was extracted from the cell pellet with MP Biomedicals Fast Soil DNA Kit. Sequencing of the 16S rRNA gene was performed using the Zymobiomics Quick 16S NGS Plus Library Prep Kit. This kit prepares V3V4 16S rRNA library; primer Plate B was used. Amplification was performed following the temperature cycling described in the manufacturer's protocol. Amplicon libraries were pooled at equal volumes and purified using the Select-a-Size DNA Clean & Concentrator MagBead Kit from Zymo. The library was sequenced on an Illumina MiSeq at the University of Tennessee, Knoxville, Center for Environmental Biotechnology using MiSeq v3 600 Cycle Kit. Raw 16S rRNA sequencing reads were demultiplexed by the Illumina MiSeq. Raw 16S rRNA sequencing data is deposited in the NCBI SRA under Bioproject PRJNA887454.

### 16S rRNA Data Analysis

To process raw sequences, we used the DADA2 (divisive amplicon denoising algorithm) package in R (Callahan et al., 2017) to trim primers, overlap paired-end reads, remove low-quality reads, and remove internal standard (phiX). Amplicon sequence variants (ASVs) were inferred. Using DADA2, denoised reads were merged, bimeric reads were removed, and then the taxonomy of ASVs was assigned using the Silva database v138. The complete pipeline, including parameters used for our analysis, can be found at https://github.com/lgschaer/full_CDPET. Analysis of sequences was performed in R (R Core Team, 2013), ver. 4.1.3. Quality control blanks were removed from the data set. Sample “L26” (LS2_Calumet, BH medium, DCPET as the carbon source, replicate two) was removed from the data set due to suspected contamination. Diversity analysis was performed using the phyloseq package (McMurdie & Holmes, 2013). Our sequences were rarefied using the “rarefy even depth” function in phyloseq to a minimum sequence sample size of 64 262 reads. Mitochondrial DNA and chloroplasts were removed from our dataset before downstream analysis was performed, yielding a minimum sequence sample size of 64 147 reads. Alpha diversity is used to measure the number of distinct species in a sample. Two common alpha diversity metrics are observed ASVs and Shannon diversity. Observed ASVs are an indication of richness (number of unique microbial taxa), and Shannon diversity accounts for the number of unique taxa and relative proportions of each microbial group (evenness). On the rarified table, alpha diversity was measured using the “estimate richness” function in phyloseq; the metrics Shannon and observed ASVs were used. To determine whether there was a significant difference in alpha diversity (richness and evenness) between each microbial consortium, a Kruskal–Wallis test was performed using the FSA (Ogle et al., 2020) package in R (*n* = 12 for each group). Due to our relatively small number of samples, Kruskal–Wallis was used because of its compatibility with non-normally distributed data and imbalanced datasets. Base R (R Core Team, 2013) was used to perform a Dunn test (post-hoc) to determine between which samples there was a significant difference. We also used phyloseq to examine differences in the microbial community composition between different enrichments and experimental treatments. A principal coordinates analysis (PCoA) plot was used to visualize these differences using a unifrac distance matrix. A permutational multivariate analysis of variance (PERMANOVA) was performed using the “adonis” function in the vegan package (Dixon, 2003) to look for statistically significant differences in community composition between pairwise comparisons of the three microbial consortia. Differential abundance analysis was performed using the DESeq2 package in R (Love et al., 2014).

### Growth on Other Deconstructed Materials

To test whether microbial consortia could degrade additional ammonium hydroxide-treated polymers, additional growth tests were performed. Two microbial communities were chosen for additional testing: LS1_Calumet and EB9_Mackinac. EB9_Mackinac is a lower diversity community that was derived from the EB2_Mackinac community used for earlier experiments. The community is further described in the supplemental materials (Supplementary Fig. 10). These communities were grown on a variety of deconstructed materials (100% nylon, 92/8% nylon/spandex, 95/5% polyester/spandex, and 100% polyester), polycarbonate, polyurethane [horizontal tubular (batch) and Parr reactors], and multilayerd plastic (Mylar) before and after extraction of polyolefin layers) to test the potential of our communities to degrade additional materials that have been chemically deconstructed. The details of the chemical deconstruction methods for the additional materials are fully described in the supplemental materials.

To measure the growth of consortia on the additional deconstructed materials, 250 μL of neutralized product was diluted in 4.75 mL of sterile water. The 96-well culture plates were set up with 180 μL of diluted deconstructed product per well. The EB9_Mackinac and LS1_Calumet cultures were used to inoculate four replicate wells on the prepared culture plates with 20 μL of culture for each deconstructed material. For each type of deconstructed material, four uninoculated blanks were used as a control. Plates were sealed with parafilm and incubated at room temperature on an open-air shaking incubator at 200 rpm. OD_600_ was measured daily using a Synergy LX (Biotek).

## Results

### Ammonium Hydroxide Efficiently Depolymerizes PET Into Soluble Monomers

Ammonium hydroxide (NH_4_OH) simultaneously depolymerized and solubilized PET (Arai et al., 2010; Zenda & Funazukuri, 2008). Recycled PET pellets (0.25 g PET/mL NH_4_OH) were processed at 240 °C for 1 hr in a custom vertical reactor, achieving 87.3 ± 9.3% solubilization (*n* = 15 batches). The liquid derived from the recycled PET contained high concentrations of the aromatic monomers terephthalic acid (44.0 g/L) and terephthalic acid monoamide (24.0 g/L) (Supplementary Fig. 1). This liquid was used as the nutrient source for bioconversion studies. There were no peaks detected corresponding to terephthalamide, MHET [mono(2-ethylhydroxyl) terephthalic acid], or BHET [bis(2-ethylhydroxyl) terephthalic acid]. As it is unlikely that there would be larger oligomers present if the dimer (MHET) and trimer (BHET) were not present, this suggests that the liquid product used for bioconversion studies contained only monomers derived from the deconstruction of PET.

### DCPET Contains Sufficient Nutrients to Support Microbial Growth in BH Medium, Scavenged River Water, and Ultrapure Water

**Fig. 1:**
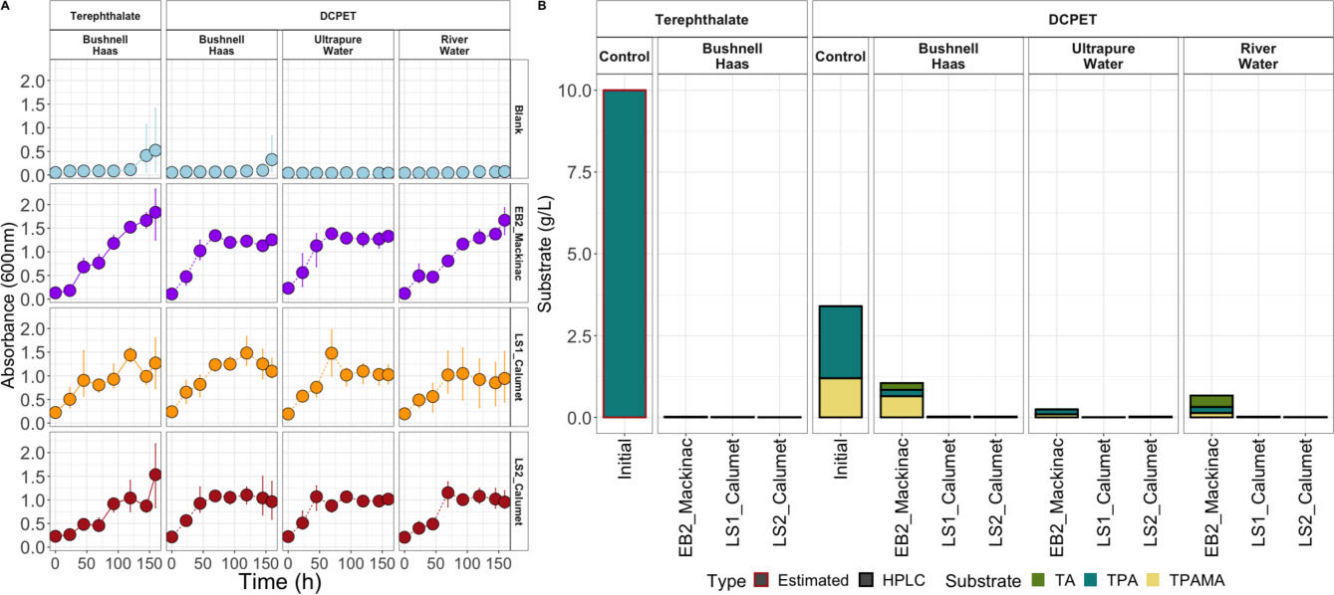
(A) OD600 of each carbon source and media type shows that the enrichments grew similarly regardless of media and carbon source. Triplicate cultures were incubated on magnetic stir plates at room temperature, stirring at 130 rpm with a Teflon-coated magnetic stir bar. (B) high-performance liquid chromatography substrate quantification for each enrichment, substrate, and media type at the end of the experiment (169 hr). Each bar represents a single sample. Estimated initial concentration for terephthalate (red outline) is based on the weight of terephthalate added to the Bushnell–Haas. DCPET = deconstructed polyethylene terephthalate; TPA = terephthalic acid; TPAMA = terephthalic acid monoamide; TA = terephthalamide.

Three microbial consortia were enriched from either agricultural compost (LS1_Calumet and LS2_Calumet) or lake sediments (EB2_Mackinac). These consortia were able to grow to high-optical densities on chemically DCPET with and without added nutrients (Fig. 1A). All cultures reached OD_600_ of greater than 0.8 over the course of seven days when grown on DCPET or the model compound terephthalate (Fig. 1A). All three consortia were able to grow to high densities when using the model compound terephthalate as the sole carbon source in the standard minimal BH medium, which contains ammonium, nitrate, and phosphate. The highest mean OD_600_ values for all conditions observed were in the EB2_Mackinac enrichments in the BH and terephthalate (mean OD_600_ 1.93 ± 0.55). Similarly, the LS2_Calumet consortia grew to very high densities on the BH and terephthalate treatments (mean OD_600_ 1.58 ± 0.68) (Supplementary Table 1). When the consortia were grown on DCPET, they grew to high densities in all the treatments (BH, autoclaved ultra-pure water, and ARW). Interestingly, for all three cultures, the highest ODs were not in the defined culture medium. For example, the LS1_Calumet and LS2_Calumet communities grew to the highest densities in the autoclaved ultrapure water (mean OD_600_ 1.01 ± 0.2 and 1.02 ± 0.01, respectively). Additionally, the highest OD_600_ reached by the EB2_Mackinac community was in the ARW treatment (mean OD_600_ 1.71 ± 0.29).

To determine if there was a difference in growth rates between the medium types, we used analysis of covariance (ANCOVA). ANCOVA results (Supplementary Table 2) showed that there was no statistically significant difference between log phase growth for DCPET treatments (autoclaved ultrapure water, ARW, and BH) for the LS1_Calumet and LS2_Calumet enrichments (Supplementary Table 2). ANCOVA results showed that the EB2_Mackinac enrichment had a statistically significant difference between log-phase growth between two treatments, however, when a Bonferroni adjusted pairwise *t*-test was performed, none of the adjusted *p*-values were statistically significant (Supplementary Table 2). Generation times and growth rates were calculated for each of the enrichments for each media and carbon type. The EB2_Mackinac enrichments had the highest growth rates, which were between 0.19 and 0.026 hr^−1^, followed by LS1_Calumet with growth rates between 0.017 and 0.018 hr^−1^, and LS2_Calumet with growth rates between 0.016 and 0.017 hr^−1^ (Supplementary Table 7, Supplementary Fig. 7).

Deconstruction of PET is expected to result in the following aromatic monomers: terephthalic acid, terephthalic acid monoamide, and terephthalamide. HPLC analysis of the expected monomers showed a starting concentration of 3.4 g/L of monomers in the media amended with DCPET. Of this, approximately 60% was terephthalic acid and one-third was terephthalic acid monoamide, with no terephthalamide detected. The LS1_Calumet and LS2_Calumet cultures degraded nearly all the DCPET (>99.1%) within seven days, with the concentration of aromatic monomers being below the detection limit (<0.01 mg/L). The three aromatic compounds were detected at below the detection limit for all media types. In contrast, the EB2_Mackinac cultures were less efficient, and while they were able to consume all of the terephthalate in the control sample, they only degraded 65–93% of the starting monomers from the DCPET. The EB2_Mackinac cultures degraded most of each substrate, but remaining terephthalate, terephthalamide, and terephthalate monoamide were detected (sum of all three compounds between 1.05 g/L and 0.24 g/L). Interestingly, terephthalamide was detected in the final samples, but not in the initial sample. Terepthalamide was detected at the highest concentration in the river water treatment for the EB2_Mackinac culture (0.35 g/L), despite there being very little terephthalamide detected in initial samples. It is possible that a similar compound was found in the river water, making this estimate artificially high. It is also possible that the cells in the culture produce a similar compound that detected by the HPLC.

Despite the BH with terephthalate cultures consuming more disodium terephthalate, this culture only grew to marginally higher-optical densities compared to the cultures amended with DCPET. Since the DCPET contains added nitrogen and phosphorus from the chemical deconstruction and neutralization, we determined the amount of nutrients added to the cultures grown in autoclaved ultrapure water and ARW. The measurements represent the starting concentrations in each experimental condition used for these measurements (Table 1). The treatments containing DCPET had higher concentrations of ammonium, dissolved organic carbon, soluble reactive phosphorus, and total dissolved nitrogen compared to the corresponding treatment without DCPET. The treatments with DCPET contained between 2.75–2.9 g N/L of ammonium and 4.4–5.5 g P/L of SRP.

**Table 1.** Media Containing Deconstructed Polyethylene Terephthalate (DCPET) Contained Sufficient Nitrogen and Phosphorus Concentrations to Support Microbial Growth Compared to Bushnell–Haas Minimal media. Ranges of Nutrient Concentrations for DCPET-Amended Solutions Were Determined From the 1:100 000 and 1:1 000 000 Dilution of the Samples

		Ammonium (mg N/L)	Dissolved organic carbon (mg C/L)	Nitrate and nitrite (mg N/L)	Soluble reactive phosphorus (mg P/L)	Total dissolved nitrogen (mg N/L)
Bushnell–Haas	With TPA	11.6	635.4	181.0	381.5	204.6
	With DCPET	2 762–2 899	11 742–37 910	156 to ∼0	4 363–5 469	3 147–5 312
Ultrapure water	No DCPET	<0.01	0.22	<0.01	<0.01	<0.01
	With DCPET	2 750–2 888	11 106–37 275	<0.01	3 981–5 088	2 942–5 108
River water	No DCPET	0.03	3.36	0.06	0.05	0.25
	With DCPET	2 750–2 888	11 106–37 275	0.06	3 981–5 088	2 942–5 108

### Microbial Communities are Dominated by Known Aromatic-Degrading Taxa

**Fig. 2:**
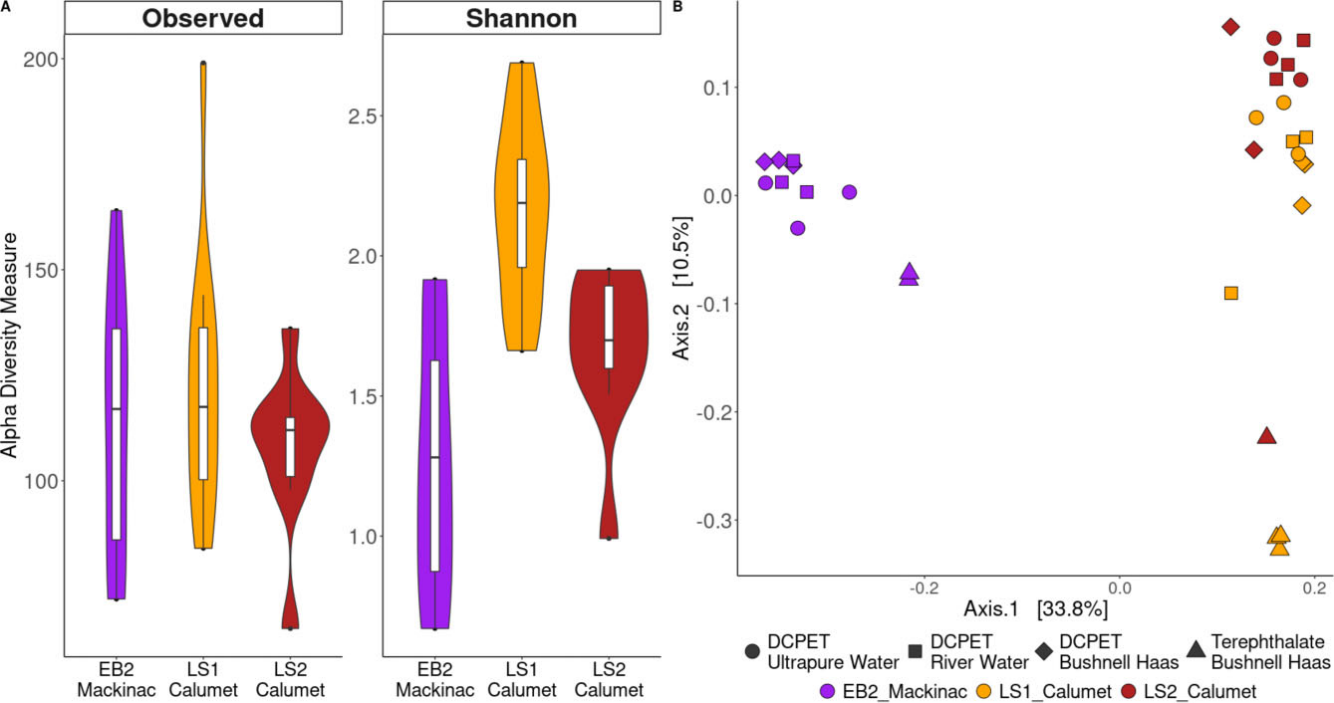
(A) Alpha diversity metrics observed amplicon sequence variants and Shannon diversity for each of the three enrichments; (B) Principal coordinates analysis plot showing the difference in community composition for EB2_Mackinac, LS1_Calumet, and LS2_Calumet by enrichment type and substrate. There were significant differences in the alpha diversity and community composition between the three enrichments.

One of the goals of this study was to determine the potential for microbial communities to be used in PET processing. To better understand the nature of the microbial communities in the consortia metabolizing the DCPET, we used 16S rRNA sequencing to profile the community composition in each media type. The 16S rRNA sequencing data was grouped into ASVs that represent unique sequences of the 16S rRNA gene. Overall, the LS1_Calumet enrichment had the highest mean alpha diversity for both metrics (Shannon 2.2 and observed 122.5). The mean alpha diversity metrics for EB2_Mackinac (Shannon 1.2 and observed 112.7) and LS2_Calumet (Shannon 1.7 and observed 107.4) show that the EB2_Mackinac enrichment had more species richness while the LS2_Calumet enrichment had higher evenness. Since samples were not normally distributed and the sample size was small, we used a Kruskall-Wallis statistical test to evaluate if there was a statistically significant difference in diversity between the three enrichments for both diversity metrics. The Kruskall–Wallis test for observed ASVs was non-significant (*p*-value .599, 2 degrees of freedom, chi-squared 1.022), however, there was a significant difference in Shannon diversity between enrichments (*p*-value < .001, 2 degrees of freedom, chi-squared 16.656). A Dunn post hoc test for Shannon diversity was used to determine between which enrichments there was a statistically significant difference (Fig. 2A, Supplementary Table 4). We found that the alpha diversity was significantly different between EB2_Mackinac and LS1_Calumet (adjusted *p*-value < .001) and LS1_Calumet and LS2_Calumet (adjusted *p*-value .022), but non-significant between EB2_Mackinac and LS2_Calumet. The same statistics were used to look for statistically significant differences in the alpha diversity between media and carbon types for each enrichment. No statistically significant differences in observed ASVs were found between media and carbon types within LS1_Calumet, LS2_Calumet, or EB2_Mackinac (*p*-values .293, .656, and .251, respectively). Similarly, no statistically significant differences were found in Shannon diversity between media and carbon types for LS1_Calumet, LS2_Calumet, or EB2_Mackinac (*p*-values .715, .133, and .099, respectively) (Supplementary Fig. 2).

To determine if the medium type or substrate was selected for a distinct community composition, we visualized community composition using PCoA of Bray–Curtis dissimilarities. PCoA showed the samples clustered most strongly by enrichment type, with the EB2_Mackinac culture clustering on the far left side of the plot and the LS1_Calumet and LS2_Calumet cultures clustering closer together on the right side of the plot (Fig. 2B). Additionally, each of the terephthalate samples clustered together separately from the DCPET samples for each enrichment, with the effect more pronounced for the Calumet enrichments. A PERMANOVA analysis showed a statistically significant difference between all three enrichments [*p*-values were .001 (EB2_Mackinac vs. LS1_Calumet and EB2_Mackinac vs. LS2_Calumet) and 0.002 (LS1_Calumet vs. LS2_Calumet), Supplementary Table 5]. Additional pairwise PERMANOVA tests showed that there was no statistically significant difference in the community composition of samples grown on each combination of media and carbon types within each enrichment (Supplementary Table 6).

**Fig. 3:**
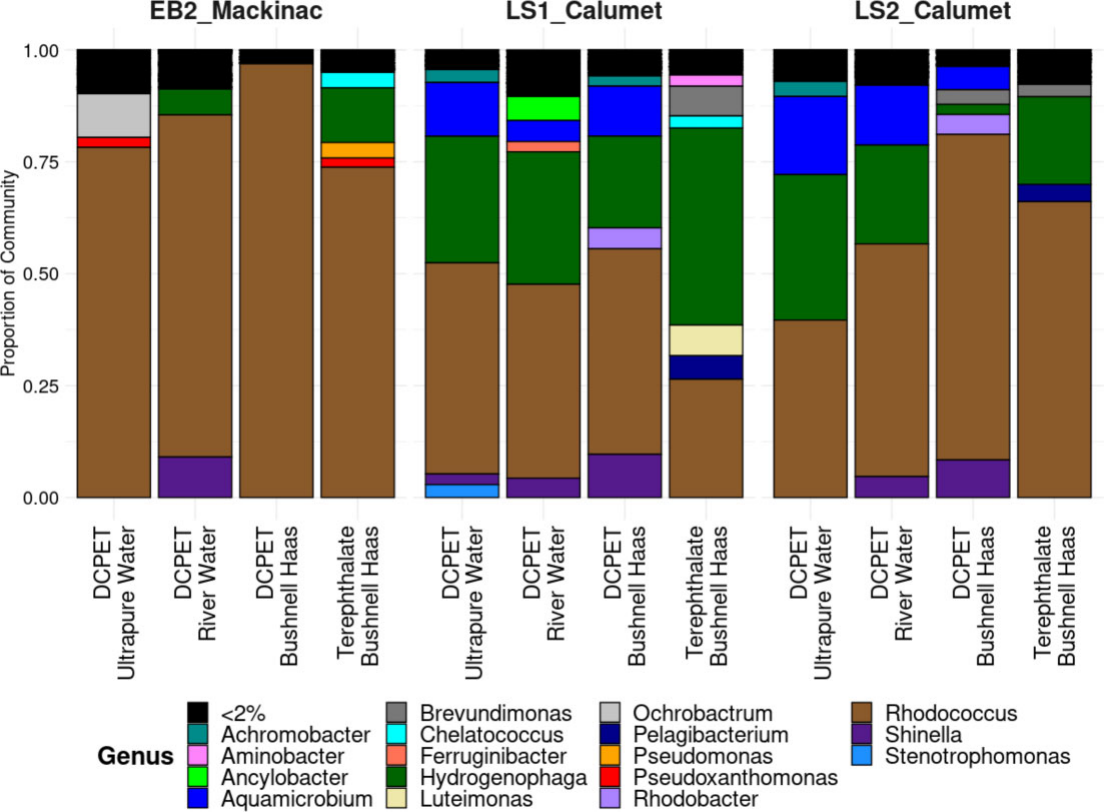
Taxonomic composition of each enrichment, media, and carbon type for genus-level classification.

**Fig. 4:**
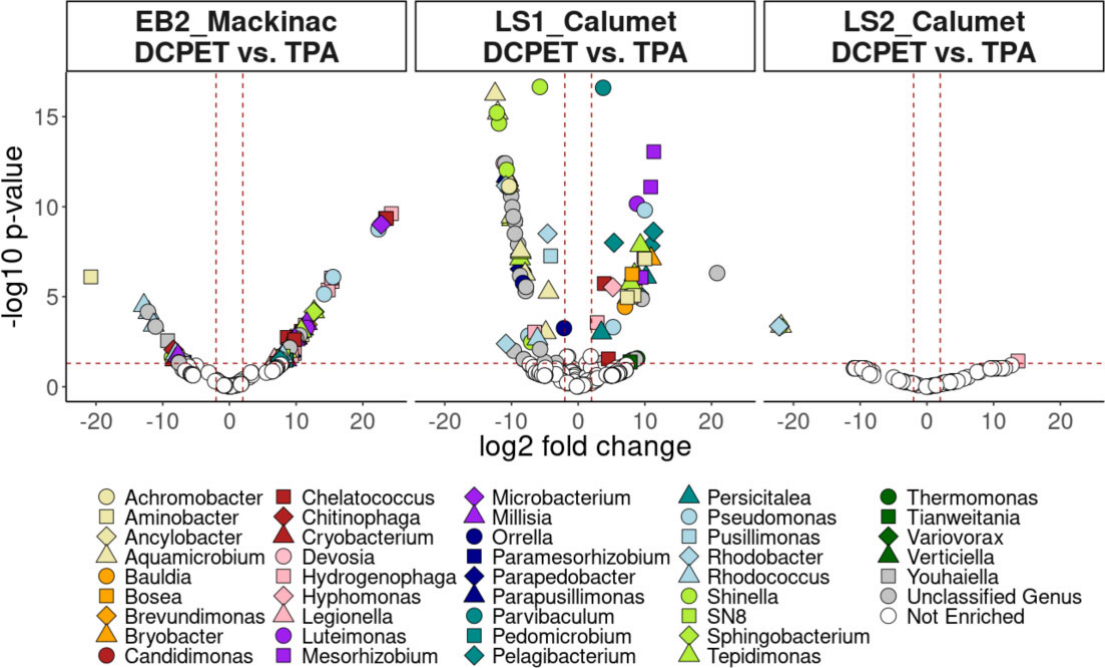
Differential abundance of ASVs in either Bushnell–Haas DCPET or Bushnell–Haas TPA cultures for EB2_Mackinac, LS1_Calumet, and LS2_Calumet. For each ASV, if log2foldchange is negative, that ASV was enriched in DCPET; if positive, the ASV was enriched in TPA. The dashed red lines represent significance cutoffs for the analysis. The horizontal line represents the –log10 of the *p*-value (.05), and the vertical lines represent the significance cutoff for the log2fold change (absolute value of 2). Colored points represent statistically significant differentially abundant organisms. TPA = terephthalate; DCPET = chemically deconstructed PET; ASVs = amplicon sequence variants.

These differences in community composition can be observed at the phylum taxonomic level. The communities within these enrichments were dominated by the phyla Actinobacteriota and Proteobacteria, with lower abundances of Acidobacteriota, Bacteroidota, Deferribacterota, Deinococcota, and Firmicutes (Supplementary Fig. 3). The EB2_Mackinac enrichments were dominated by Actinobacteriota (relative abundance 99 to 96%) with very low abundances of Proteobacteria (<0.02). The LS1_Calumet and LS2_Calumet enrichments were dominated by both Actinobacteriota (relative abundances 89 to 78% and 96 to 85%, respectively) and Proteobacteria (relative abundances 20 to 9% and 15 and 3%, respectively). These Actinobacteria were primarily *Rhodococcus* (Nocardiaceae), which made up a large proportion of the community across all enrichments (mean relative abundance 82, 41, and 54% in EB2_Mackinac, LS1_Calumet, and LS2_Calumet, respectively) (Fig. 3). *Rhodococcus* ASVs were differentially abundant in DCPET treatments for LS1_Calumet and EB2_Mackinac, and in the EB2_Mackinac TPA treatment (Fig. 4). Of the Protobacteria, *Hydrogenophaga* (Comamonadaceae) was also highly abundant across all three enrichments, particularly in LS1_Calumet and LS2_Calumet (mean relative abundance 5, 31, and 21% in EB2_Mackinac, LS1_Calumet, and LS2_Calumet, respectively) (Fig. 3, Supplementary Fig. 5). These differences in community composition help to explain the clustering that was observed in the PCoA plot (Fig. 2B).

To determine which taxa in the enrichments were affected by the substrate, we performed a differential abundance analysis using DESeq2 (Fig. 4, Supplementary Fig. 8). The most differentially abundant ASVs in the DCPET treatment compared to the terephthalate treatment for the LS1_Calumet and LS2_Calumet enrichments were *Hydrogenophaga* and *Pseudoxanthomonas* ASVs. *Hydrogenophaga, Tepidimonas*, and *Millisia* were enriched in the DCPET treatment for the EB2_Mackinac enrichment relative to the terephthalate treatment (Fig. 4, Supplementary Fig. 8). Additionally, *Shinella* and *Aquamicrobium* were significantly enriched in the DCPET treatments for the LS1_Calumet enrichment. The most differentially abundant ASVs in the terephthalate treatment for the EB2_Mackinac enrichment belonged to unclassified genera. ASVs classified as *Parvibaculum* and *Mesorhizobium* were enriched in the terephthalate treatments for the LS2_Calumet enrichment (Fig. 4).

*Hydrogenophaga* ASVs were differentially abundant in the TPA treatments for all enrichments; one *Hydrogenophaga* ASV was differentially abundant in LS1_Calumet (Fig. 4). Other highly abundant organisms include *Aquamicrobium* (Rhizobiaceae) in LS2_Calumet (maximum relative abundance 19%); additionally, eight *Aquamicrobium* ASVs were found to be differentially abundant in the LS1_Calumet enrichment in the DCPET treatment. *Ochrobactrum* (Rhizobiaceae) was highly abundant in EB2_Mackinac (maximum relative abundance 14%), and *Shinella* (Rhizobiaceae) in EB2_Mackinac, LS1_Calumet, and LS2_Calumet (maximum relative abundances 14, 11, and 9%, respectively). *Shinella* ASVs were differentially abundant in the DCPET treatments for all enrichments. In addition, *Luteimonas* and *Ancylobactor* (Xathomonadaceae) were both found in LS1_Calumet (maximum relative abundances 17 and 16%, respectively). *Ancylobacter* ASVs were differentially abundant in the terephthalate treatments for LS1_Calumet and LS2_Calumet, and *Luteimonas* ASVs were found to be differentially abundant in the DCPET treatments for all three enrichments.

### Microbial Communities Grow on a Variety of Deconstructed Polymers

**Fig. 5:**
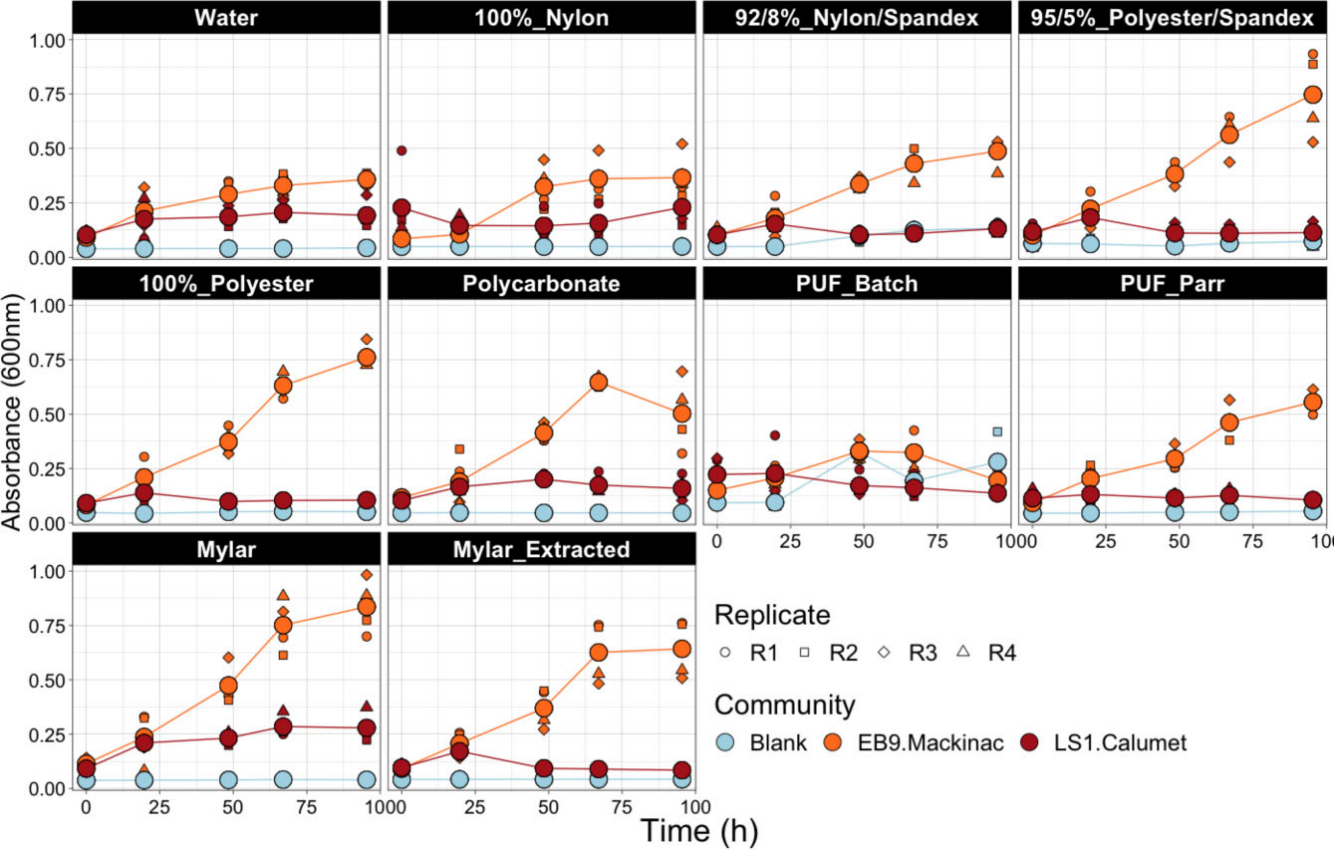
Growth of select communities on additional deconstructed materials in ultrapure water. Each culture had four replicates and was incubated at room temperature in a 96-well culture plate. The EB9_Mackinac community grew on all substrates except for the polyurethane foam batch (PUF batch). The LS1_Calumet community grew to lower densities, but showed some growth on Mylar, Polycarbonate, and 100% Nylon. PUF_Batch = polyurethane foam in a tubular reactor; PUF_Parr = polyurethane foam in a stirred Parr reactor; Mylar = multilayer plastic-aluminum film; Mylar_Extracted = multilayer plastic-aluminum film that had been processed to remove the polyolefin layer, leaving only the polyester and aluminum.

Ammonium hydroxide treatment is also able to deconstruct a variety of other polymers, such as polyurethane foam, polycarbonate, synthetic fabrics (polyester, nylon, and spandex blends), and the polyester layer of multilayered plastics (Fig. 5, Supplementary Table 8) (Adams & Baron, 1965; Arai et al., 2010; Mormann et al., 2004). For most of these materials, between 70 and 90% of the polyester, polyamide (nylon), or polyurethane (spandex, PUF) were depolymerized under unoptimized reaction conditions. The exceptions were the polyurethane foam that did not process well in a tubular reactor and the polycarbonate shards. Following neutralization using phosphoric acid, the liquid products were largely able to support growth as the sole source of carbon, nitrogen, and phosphorus by two consortia, LS1_Calumet and EB9_Mackinac. EB9_Mackinac is a minimal community derived from the EB2_Mackinac community (Supplementary Fig. 10). The EB9_Mackinac community grew to higher densities than the water-only control on all but the 100% nylon and PUF_batch substrates; in these treatments, the growth of EB9_Mackinac was similar to that of the water-only control. Growth observed in the water-only control could be due to residual substrate transferred along with the inoculum. The lack of growth on these substrates could be due to the low concentration of carbon, low solubilization of the polymers (Supplementary Fig. 11), or low solids loading in the reactor (Supplementary Table 8) compared to the other materials. The LS1_Calumet community grew at lower densities compared to the EB9_Mackinac community, but was able to grow on the products from the Mylar, polycarbonate, and 100% nylon fabric (Fig. 5). These findings indicate that microbial consortia are capable of metabolizing diverse deconstructed polymers using only the neutralized reaction product in water as the medium.

**Fig. 6:**
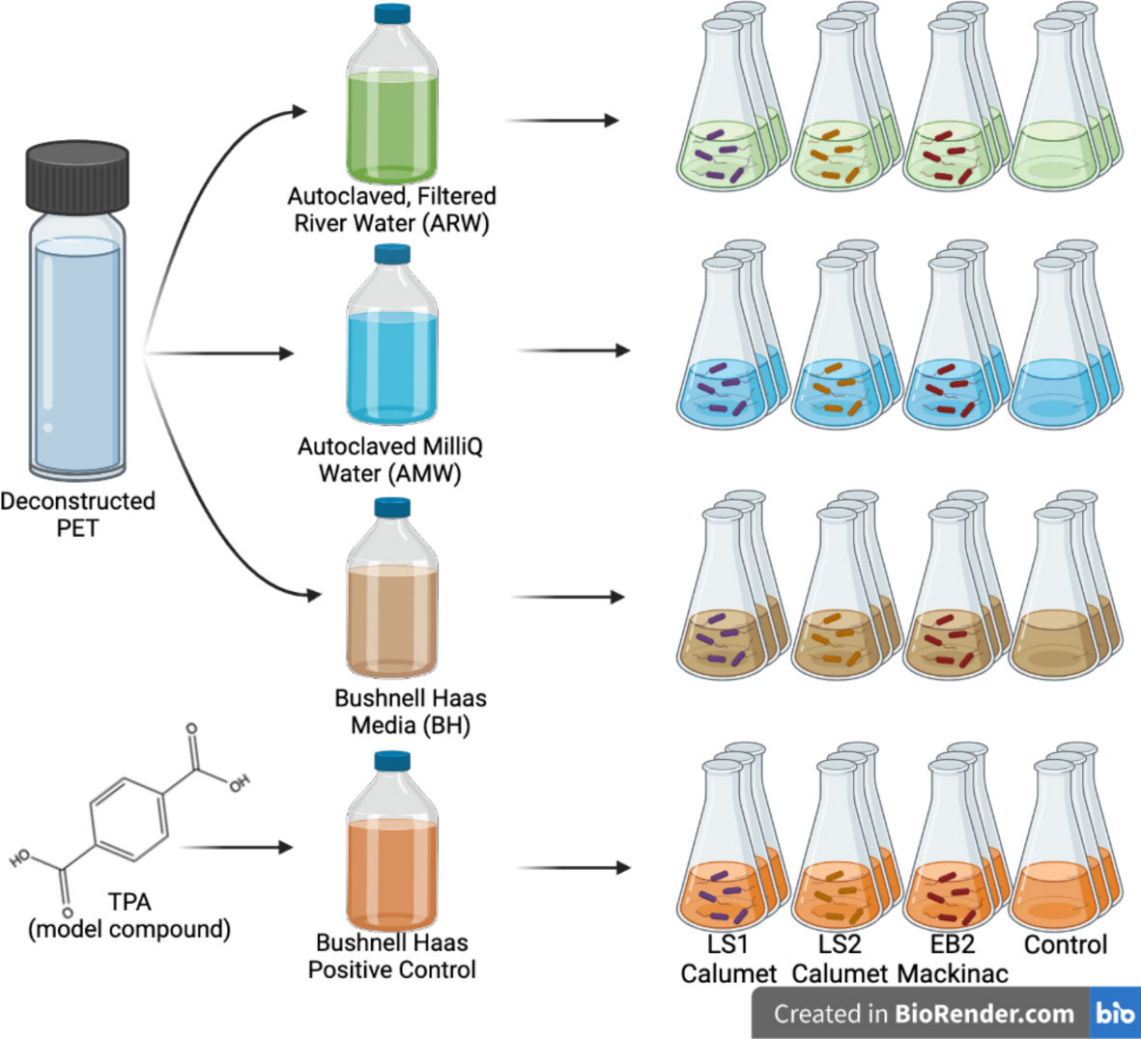
Experimental design for testing the consortial growth on deconstructed polyethylene terephthalate in culture medium, ultrapure water, and sterilized river water.

## Discussion

It is essential to develop creative solutions for dealing with the growing amounts of plastic waste. Chemical and biological methods have been proposed for recycling and upcycling of plastic wastes (Blank et al., 2020; Byrne et al., 2022; Guzik et al., 2021; Schaerer et al., 2022; Werner et al., 2021). The coupling of chemical and biological methods for plastic processing have shown to be useful in enabling rapid and tailorable plastic upcycling (Blank et al., 2020; Byrne et al., 2022; Guzik et al., 2021; Werner et al., 2021). However, most of these studies have focused on a single waste stream and isolated bacteria. Here we demonstrate a process for tandem chemical and biological processing that allows for flexible processing of diverse plastic wastes using chemical deconstruction coupled to a microbial community. The streamlined process demonstrated here is advantageous because it eliminates the need for costly growth medium and allows for flexible conversion of plastic waste into biomass through the use of microbial communities.

The chemically DCPET product produced in this study contains terephthalate, terephthalic acid monoamide, terephthalamide, and ethylene glycol as carbon sources, along with the nitrogen and phosphorus added during the chemical deconstruction process. In this study, we grew microbial consortia on DCPET in three medium types: autoclaved ultrapure water, sterilized river water, and BH medium. No statistically significant difference was found in covariance of the relationship between OD_600_ and time for each enrichment growing on DCPET in each of these three types of media. This suggests that BH media did not provide an advantage for growth compared to scavenged water or ultrapure water. This was supported by the measured nutrient content of the different media types, which showed an abundance of nitrogen and phosphorus in the media containing DCPET, well beyond what was provided in the BH media. This indicates that there is room to reduce the ammonium hydroxide concentration and phosphorus loadings while still maintaining comparable cell growth. Because the starting inoculum for this experiment were grown on terephthalate in BH medium, there is potential that some additional nitrogen and phosphorus was carried into the experimental samples along with the inoculum. However, we expect any carry-over of nitrogen and phosphorus to be negligible compared to the nutrient concentrations in the DCPET amended treatments (Table 1).

Our results show that natural microbial communities enriched from different environments to degrade terephthalate can also degrade the neutralized product of DCPET. Two of the consortia from this study (LS1_Calumet and LS2_Calumet) were able to metabolize 99.8% of the phenolic monomers from PET degradation. These results support previous research, which have demonstrated that combined chemical and biological recycling methods may be a feasible option for upcycling of excess PET waste (Werner et al., 2021). Together, the microbial consortia achieved between 57.1 and 99.8% of the deconstructed monomers over the course of seven days. Previous reports have shown that over the course of 6 weeks *I saikaiensis* will metabolize the majority of PET films (Son et al., 2019). The work presented here supports previous studies that have demonstrated that coupled chemical and biological processing of PET greatly increases the rates of PET processing relative to *I. sakaiensis.* Our work also demonstrates that bacterial communities capable of degradation of deconstructed plastics are common in the environment. We were able to obtain consortia from compost and lake sediment. Neither of these sites had evidence of previous PET contamination, which suggests that terephthalate degradation may be a common microbial metabolism.

The consortia that we derived were able to rapidly metabolize DCPET. To better understand the differences observed in the extent of DCPET degradation, we used 16S rRNA sequencing to profile the community composition. One weakness of the work presented here is the lack of genomic information for these consortia. Future work could explore the genomes of these consortia to further evaluate their biodegradation potential and identify primary degraders within the microbial communities. However, many of the genera identified in this study are closely related to organisms known to degrade a variety of environmental contaminants. We found that the enrichments for LS1_Calumet and LS2_Calumet were dominated by both *Hydrogenophaga* spp. and *Rhodococcus* spp., while the EB2_Mackinac enrichments were primarily dominated by *Rhodococcus* spp. *Rhodococcus* spp. are known to degrade PAHs (Wang et al., 2021), phthalates (Choi et al., 2005), and lignin (Bhatia et al., 2019). *Rhodococcus erythropolis HX7* was isolated from Russian soil and is known to degrade oil (Novikov et al., 2021). *Rhodococcus jostii* RHA1 is a known phthalate and aromatic degrader and was used to identify the terephthalate degradation pathway (Hara et al., 2007). *Hydrogenophaga* spp. have been shown to degrade aromatic compounds: *H. aromaticiuorans* degrades para- and meta-xylene and benzene (Banerjee et al., 2019); *H. intermedia* strain PCB syntrophically degrades aminobenzene sulfonate with a *Ralstonia* spp. (Gan et al., 2017); *Hydrogenophaga* sp. PYR1 produces surfactants that aid in the degradation of pyrene and benzo[a]pyrene (Yan et al., 2017). An enzyme isolated from *Hydrogenophaga* sp. PML113 possessed MHETase activity, and there is evidence that this species possesses genes for terephthalic acid catabolism (Knott et al., 2020).

Many of the taxa that were differentially abundant in either our DCPET or terephthalate treatments have been previously identified as organisms, which break down PET and PET derivatives (such as terephthalate). The consortia derived in our study contained many taxa that have been previously shown to degrade terephthalate, including *Rhodococcus* spp. (Auta et al., 2018; Choi et al., 2005; Hara et al., 2007), *Comamonadaceae* spp. (Knott et al., 2020; Sasoh et al., 2006), and *Pseudomonas* spp. (Roberts et al., 2020). In addition to known PET-degrading organisms, many of the differentially enriched genera in our experiment are known to degrade a wide variety of other recalcitrant aromatic compounds. Pereyra-Camacho et al. observed a consortia of saline soil bacteria with *Achromobactor* spp., *Pseudomonas* spp., and *Brevundimonas* spp. (Pereyra-Camacho et al., 2021) degrading diisononyl phthalate, a common plasticizer; these three genera were identified in our study*. Achromobactor denitrificans* PR1 is a known sulfonamide degrader that has been isolated from wastewater activated sludge, although this ability does not seem widespread in other *Achromobactor* spp. isolates (Deng et al., 2016). *Aminobactor* sp. MSH1 degrades 2,6-dichlorobenzamide (2,6-DCBA) (Albers et al., 2014; Horemans et al., 2017; T'Syen et al., 2018) using the *bbdD* gene that encodes for an aromatic ring hydroxylating dioxygenase alpha subunit which assists in the breakdown of 2,6-DCBA (T'Syen et al., 2018). The *bbdD* gene is also found in *Pusillimonas noetemanni* (T'Syen et al., 2018). Interestingly, *Pusillimonas* spp. in our experimental samples were found to be differentially enriched when the LS1_Calumet enrichment was grown on terephthalate. *Brevundimonas* spp. isolated from wastewater were previously shown to degrade quinolin (Wang et al., 2015), as well as synthetic lubricating oil (Basuki, 2017). *Brevundimonas* sp. QPT-2 hydrolyzes a wide range of aromatic herbicides using a carboxylesterase to produce alcohol and carboxylate (Xu et al., 2019). A network analysis of polycyclic aromatic hydrocarbon-degrading microbial communities showed that a *Chelatococcus* sp. was one of the most abundant organisms and had many positive interactions with other organisms (Patel et al., 2021). A few organisms from the genera *Hyphomonas* and *Luteimonas* also degrade polycyclic aromatic hydrocarbons (Dong et al., 2015; Singleton et al., 2016). Some of the differentially enriched genera in our study participate in polychlorinated biphenyl degradation, particularly *Chitinophaga* and *Mesorhizobium* (Teng et al., 2016; Weiland-Bräuer et al., 2017). Another study found that *Flavobacterium* spp. and *Pseudomonas* spp. isolated from petroleum-contaminated soils degraded many aromatic pollutants including benzene, hexane, toluene, naphthalene, and xylene (Hemalatha & Veeramanikandan, 2011). Several *Aquamicrobium* spp. are thought to contribute to the degradation of aromatic contaminants: An *A. soli* sp. NK8T was isolated from chlorobenzoate-contaminated soil (Xu et al., 2017), *A. terrae* was isolated from contaminated soil near a chemical factory (Wu et al., 2014), *Aquamicrobium* strain SK-2 was isolated from sewage sludge and is thought to use carbon from polychlorinated biphenyl to support its growth (Chang et al., 2015), and *A. defulvii* st. W13Z1 was isolated from petroleum-contaminated drill cuttings (Wang et al., 2015). Finally, the genus *Shinella* has a wide range of functional diversity from nitrogen fixation to degradation of recalcitrant pollutants. This genus has been observed degrading nicotine, 4-aminobenzenesulfonate, chloronathanil, pyridine, and 1H-1,2,3-triazole (Wang et al., 2021; Wu et al., 2016).

Differential abundance analysis showed many ASVs enriched in either BH and DCPET or BH and terephthalate treatments. It is possible that the ASVs enriched in the BH and terephthalate treatments do not grow well in the presence of some of the components of the DCPET, such as ethylene glycol. Both ethylene glycol and terephthalamide (Watanabe, 1991) are known to have antimicrobial properties, which may explain why some microorganisms do not grow when these compounds are present. It is possible that the ASVs enriched only in the DCPET-BH treatments can grow in the presence of these compounds or may contribute to their degradation. Additionally, the BH-DCPET media contained significantly more nitrogen and phosphorus compared to the BH-terephthalate media. The diversity of taxa related to known aromatic degrading bacteria in these consortia led us to determine the ability of these same consortia to degrade additional deconstructed polymers. In addition to chemically DCPET, we also tested the ability of two additional microbial communities to grow on a variety of other deconstructed materials (100% nylon, 92/8% nylon/spandex, 95/5% polyester/spandex, 100% polyester, polycarbonate, polyurethane, and two types of Mylar) in ultrapure water without added nutrients. Previous literature and these preliminary results support the potential for future systems that combine chemical methods and biological degradation to degrade flexible inputs of different waste materials (Sullivan et al., 2022). We believe that there are many benefits to using microbial consortia, including increased flexibility and potential for division of labor. However, future studies will be needed to explore the stability of the microbial consortia to ensure that the communities do not substantially change in community composition or function over time and fluctuating conditions.

This study has primarily focused on microbial biodegradation and the subsequent conversion of deconstructed plastic into microbial biomass. Previous studies have shown the ability of bioengineered isolates to produce value-added products from deconstructed plastic waste (Liu et al., 2022; Werner et al., 2021). One disadvantage of using a microbial community to produce value-added compounds from plastic waste is our current inability to bioengineer microbial communities. Presently, the technology does not exist to bioengineer microbial communities (Moon, 2022), but it is possible that future work could artificially introduce engineered organisms into these consortia that would enable conversion of diverse inputs into value-added products; however, it may be challenging to achieve stability of the introduced organism(s). This would be an important area for future work since synthetic microbial communities that include both natural and engineered isolates are of great interest to leverage the modularity and flexibility of microbial consortia to produce specific valuable compounds (Liu et al., 2022; Moon, 2022). Furthermore, microbial biomass has been proposed as a potential alternative food source to address sustainable food production through cellular agriculture (Nyyssölä et al., 2022; Ritala et al., 2017; Schaerer et al., 2022). Therefore, there is potential that microbial biomass grown on deconstructed plastic waste feedstocks could be harvested as an alternative food source, such as single-cell protein (Schaerer et al., 2022), or used to produce nutritional compounds.

Our findings indicate chemical deconstruction with ammonium hydroxide can be utilized to deconstruct PET polymers into a mixture of monomers, which can be degraded by microbial communities without additional nutrients. In addition, the microbial communities showcased here have the flexibility to degrade the nutrient-rich products resulting from the chemical deconstruction of a variety of polymers without the need for specialized medium. The approach described here could form the basis of an industrial system capable of efficient tandem chemical and biological upcycling of mixed plastic waste using microbial consortia.

## Supplementary Material

kuad008_Supplemental_Files
