# Supplementary material for: Versatile microbial communities rapidly assimilate ammonium hydroxide-treated plastic waste
Source: J Ind Microbiol Biotechnol. 2023 Apr 14;50(1):kuad008. doi: 10.1093/jimb/kuad008 (PMC10124128; doi:10.1093/jimb/kuad008)
Supplement: kuad008_Supplemental_Files [file kuad008_supplemental_files.zip › Schaerer_etal_DCPET_12_21_2022_Supplemental.pdf]

Supplemental Materials: Versatile microbial communities rapidly assimilate ammonium hydroxide-treated plastic waste.

By Laura Schaerer, Emily Wood, M. Aamir Bashir, Sulihat Aloba, Emma Byrne, Kaushik Baruah, Elizabeth Schumann, Libby Umlor, Ruochen Wu, Hyeonseok Lee, Christopher J. Orme, Aaron D. Wilson, Jeffrey A. Lacey, Rebecca Ong, and Stephen M. Techtmann

Supplemental Materials

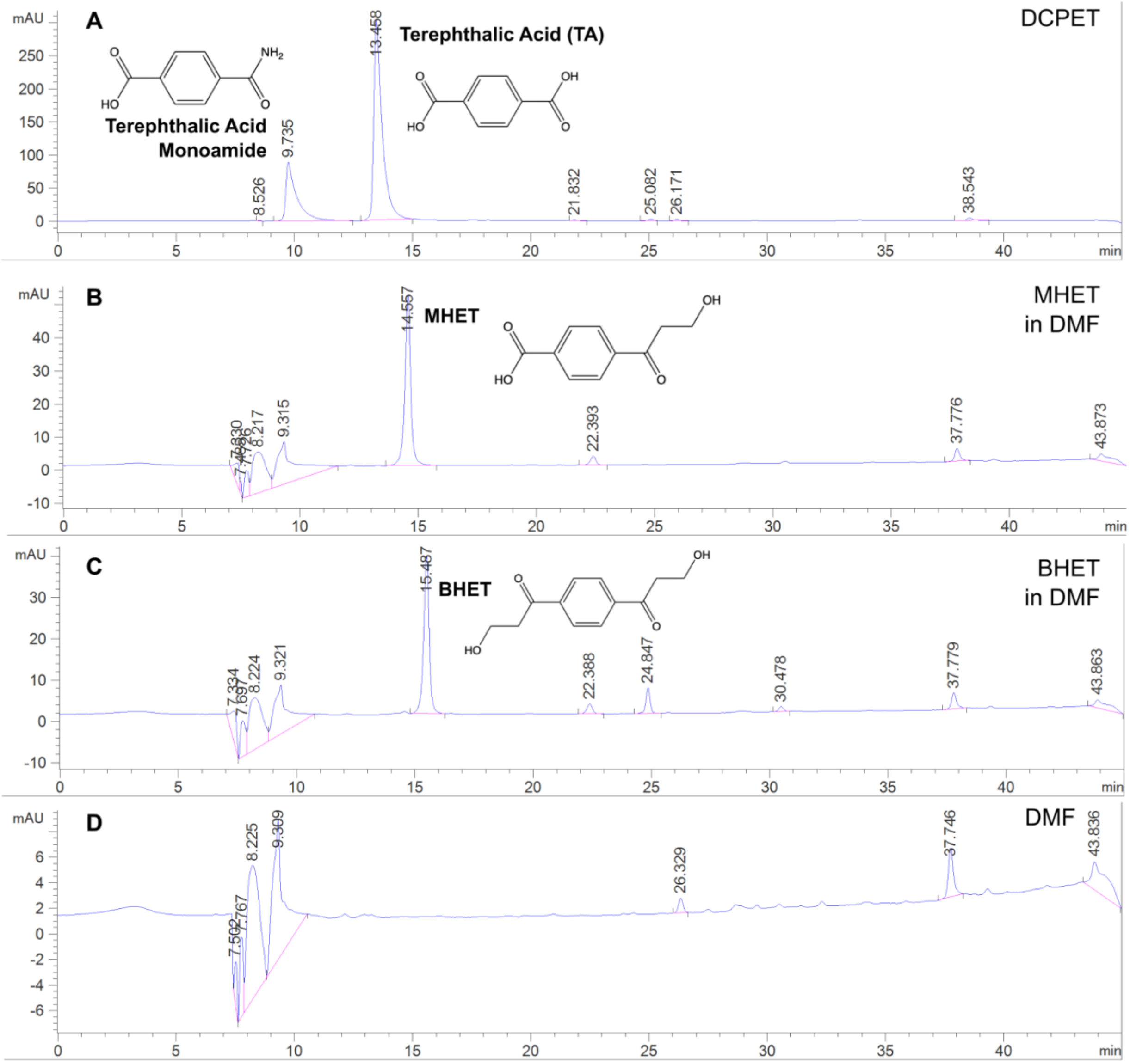

Figure S1: HPLC Chromatograms for A) deconstructed and neutralized PET liquid product, B) MHET (mono-(2-hydroxyethyl) terephthalic acid) in DMF solvent, C) BHET (bis-(2-hydroxyethyl) terephthalic acid) in DMF solvent, and D) the DMF solvent. All samples were evaluated using a diode array detector (DAD) at 300 nm wavelength.

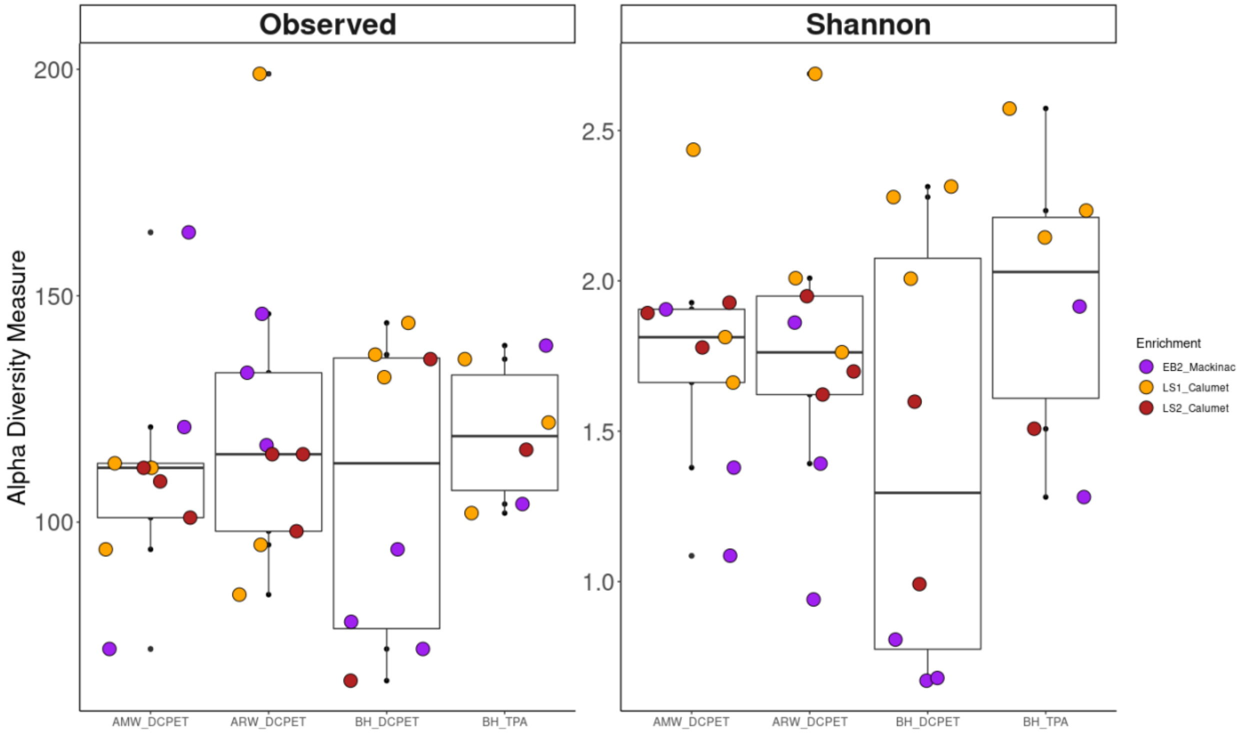

Figure S2: Alpha diversity by media type, colored by enrichment. DCPET = Deconstructed polyethylene terephthalate, TPA = terephthalate, ARW = autoclaved river water, AMW = autoclaved river water, BH = Bushnell Haas.

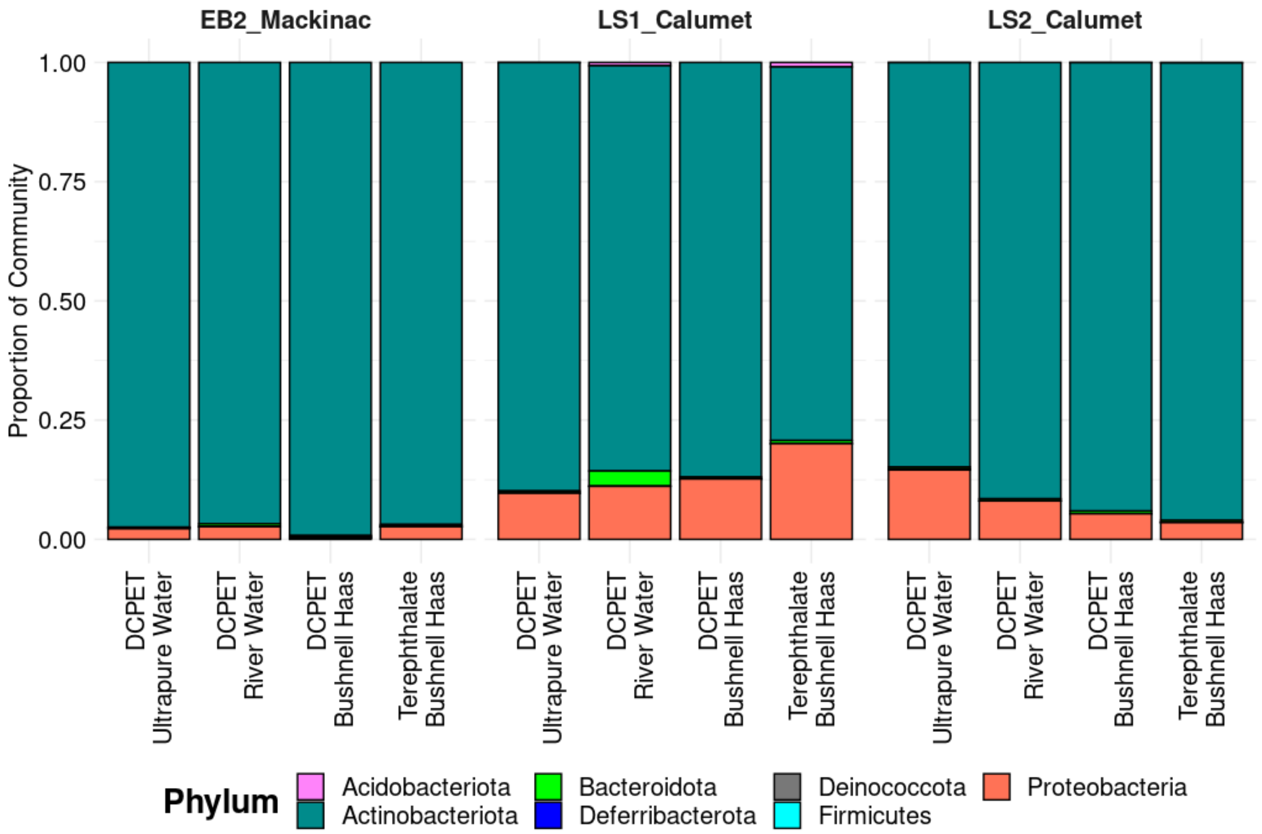

Figure S3: Phylum level community composition. DCPET = Deconstructed polyethylene terephthalate.

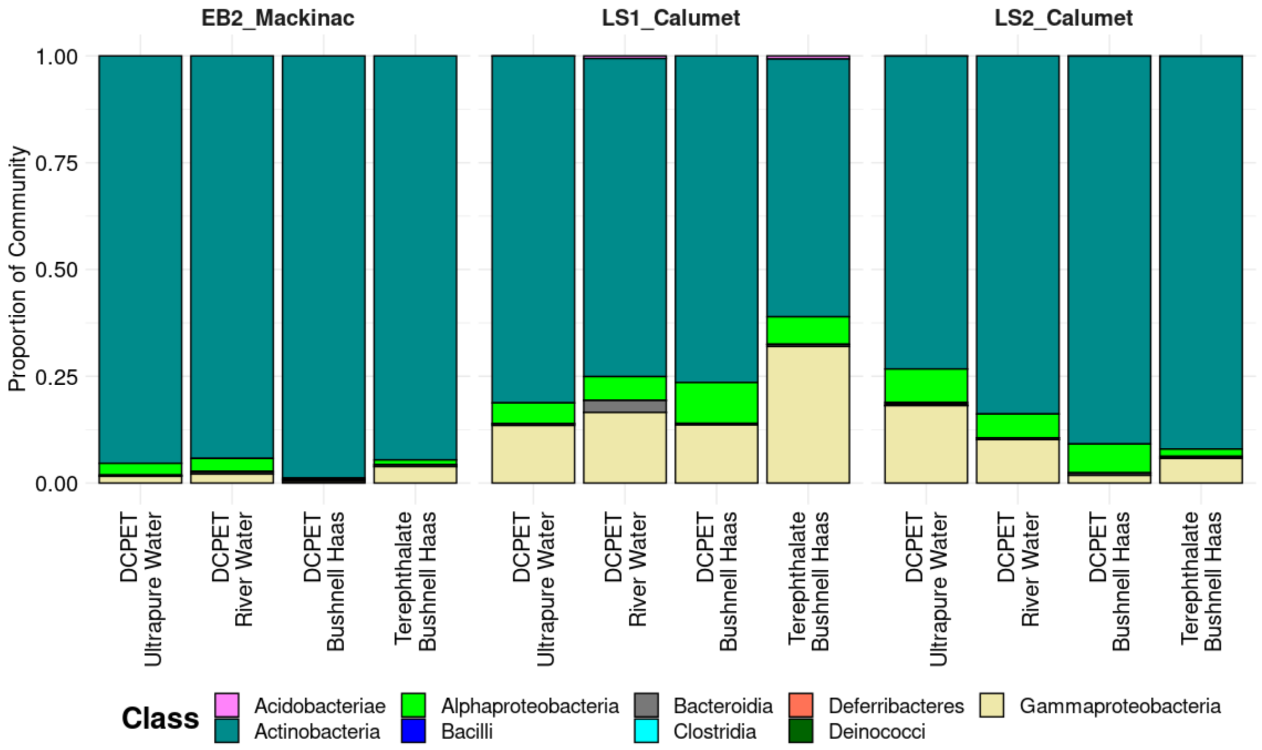

Figure S4: Class level community composition. DCPET = Deconstructed polyethylene terephthalate.

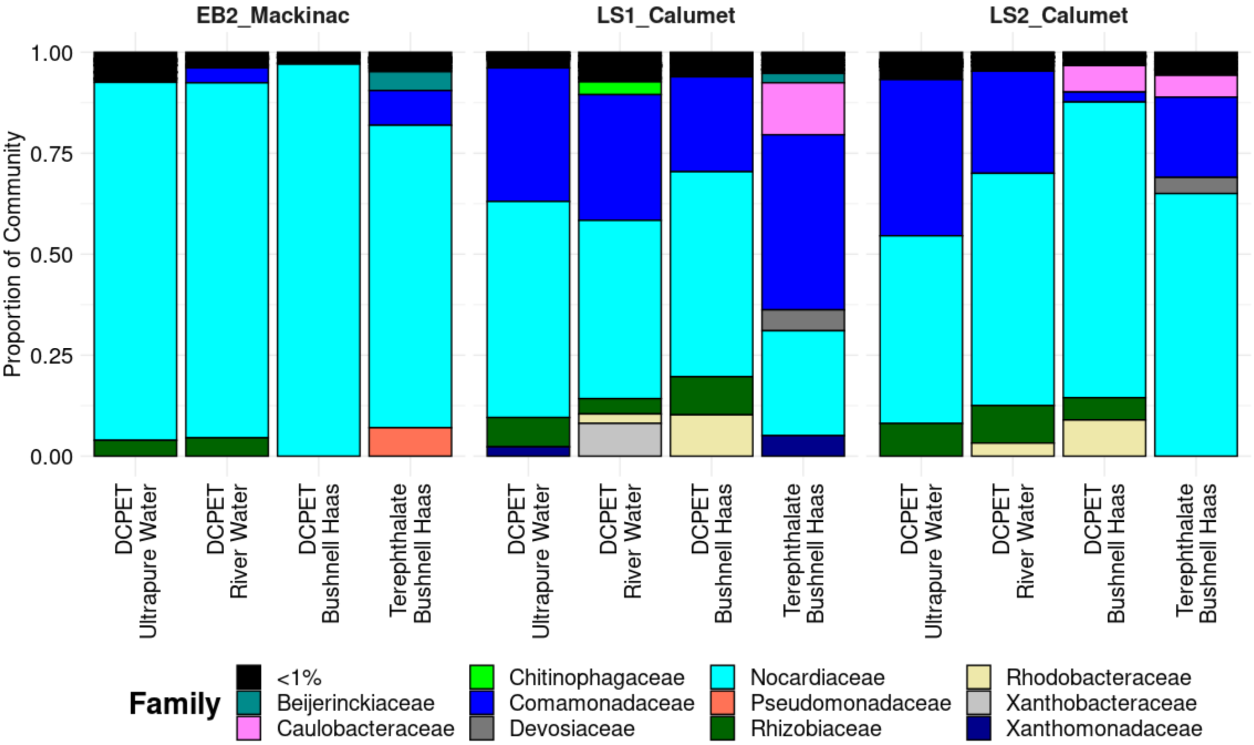

Figure S5: Family level community composition. DCPET = Deconstructed polyethylene terephthalate.

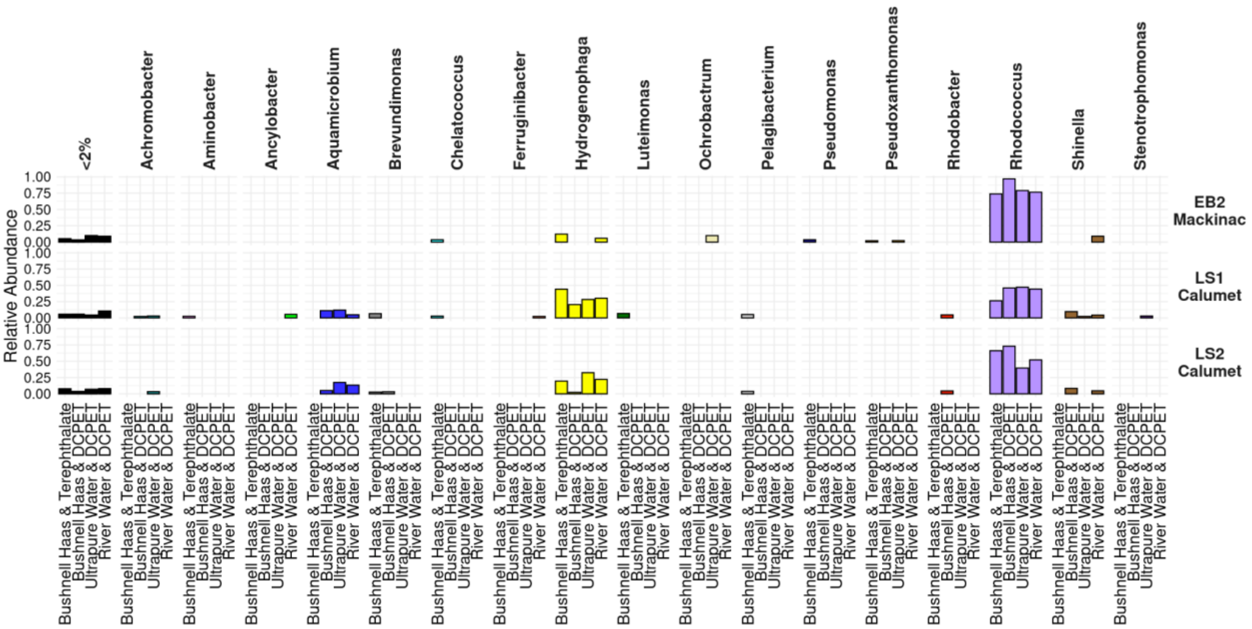

Figure S6: Relative abundance of genera in each enrichment and treatment.

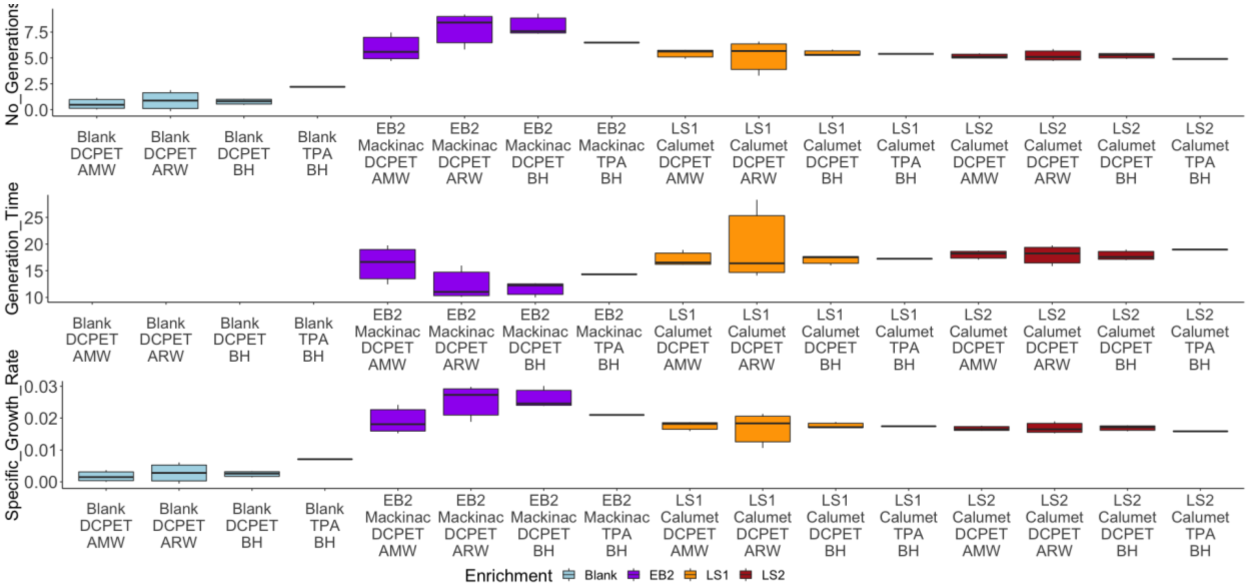

Figure S7: Number of generations, generation time, and specific growth rates for each enrichment and treatment. Calculations are based on T0 as the initial time point and T4 as the final time point. Due to very large generation times for the blanks, they have been removed from the figure. DCPET = Deconstructed polyethylene terephthalate, TPA = terephthalate, ARW = autoclaved river water, AMW = autoclaved river water, BH = Bushnell Haas.

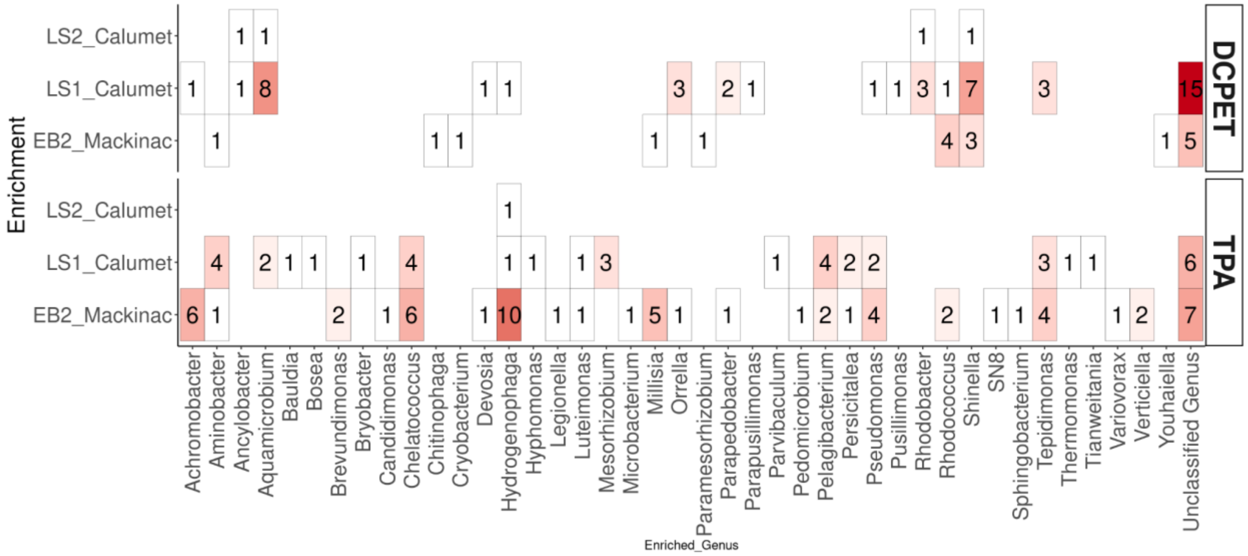

Figure S8: Number of ASVs belonging to each enrichment that were differentially abundant on each substrate, terephthalate (TPA) or deconstructed PET (DCPET) for each enrichment. Differential abundance analysis of the EB2_Mackinac cultures showed 40 ASVs enriched in the DCPET Bushnell Haas samples compared to 89 ASVs enriched in the TPA Bushnell Haas samples, the LS1_Calumet cultures had 87 ASVs enriched in the DCPET Bushnell Haas samples compared to 74 ASVs enriched in the TPA Bushnell Haas samples, and the LS2_Calumet

cultures had 29 ASVs enriched in the DCPET Bushnell Haas samples compared to 38 ASVs enriched in the TPA Bushnell Haas samples

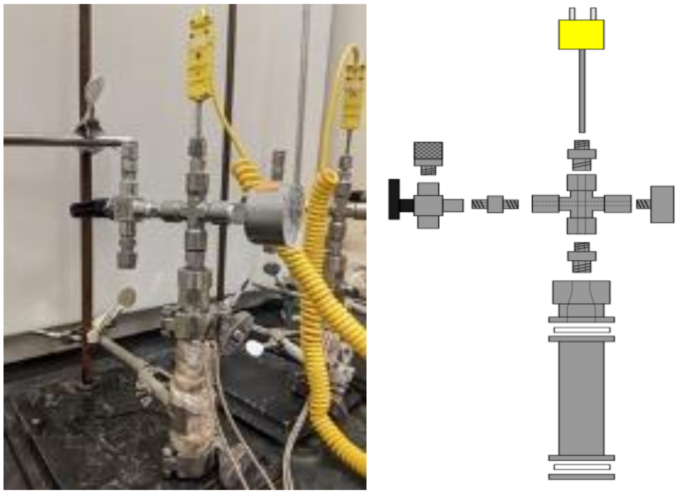

Figure S9 Design of reactors used to produce chemically deconstructed PET using aminolysis and hydrolysis.

Table S1 Final OD_{600} measurements for each enrichment, media, and carbon type. Each group represents three replicates.

Enrichment	Media	Carbon	Mean OD600 ± Standard Deviation	Maximum OD600	Minimum OD600
EB2_Mackinac	BH<br>AMW<br>ARW<br>BH	TPA<br>DCPET<br>DCPET<br>DCPET	1.93±0.55<br>1.35±0.12<br>1.71±0.29<br>1.25±0.02	2.33<br>1.44<br>1.93<br>1.28	1.25<br>1.20<br>1.36<br>1.25
LS1_Calumet	BH<br>AMW<br>ARW<br>BH	TPA<br>DCPET<br>DCPET<br>DCPET	1.28±0.54<br>1.01±0.20<br>0.88±0.54<br>0.96±0.24	1.80<br>1.23<br>1.51<br>1.38	0.73<br>0.83<br>0.45<br>0.95
LS2_Calumet	BH<br>AMW<br>ARW<br>BH	TPA<br>DCPET<br>DCPET<br>DCPET	1.58±0.68<br>1.02±0.01<br>0.85±0.21<br>0.91±0.40	2.19<br>1.03<br>1.20<br>1.39	0.84<br>1.00<br>0.82<br>0.59

Table S2 Statistical analysis of growth curves compared by DCPET treatment group for each enrichment. P-value is reported and F-value is included in parentheses.

Enrichment	Variable	ANCOVA Interaction	ANCOVA No Interaction	ANOVA
EB2_Mackinac	Time	< 0.001 (141.71)	< 0.001 (143.95)	0.513 (0.68)
	Treatment	0.002 (7.63)	0.001 (7.75)	
	Time:Treatment	0.513 (0.68)		
LS1_Calumet	Time	< 0.001 (64.78)	< 0.001 (67.62)	0.837 (0.14)
	Treatment	0.226 (1.55)	0.211 (1.61)	
	Time:Treatment	0.873 (0.137)		
LS2_Calumet	Time	< 0.001 (100.19)	< 0.001 (104.37)	0.837 (0.18)
	Treatment	0.268 (1.36)	0.254 (1.42)	
	Time:Treatment	0.837 (0.179)		

Table S3 Biodegradation of TPA and DCPET by enrichment and substrate. Units are percent of substrate degraded.

Enrichment	% degraded at T3 (69.2 hours)		% degraded at T7 (159.5 hours)	
	TPA	DCPET	TPA	DCPET
EB2_Mackinac	81.3	32.9	92.6	42.9
LS1_Calumet	83.7	68.9	92.8	74.0
LS2_Calumet	84.2	67.0	93.8	74.7

Table S4 Dunn post hoc test comparing alpha diversity of enrichments for Shannon diversity.

Comparison	Z statistic	Unadjusted p-value	Adjusted p-value
EB2_Mackinac vs LS1_Calumet	-4.022187	5.766022e-05	0.0001729807
EB2_Mackinac vs LS2_Calumet	-1.343968	1.789587e-01	0.1789587332
LS1_Calumet vs LS2_Calumet	2.437614	1.478453e-02	0.0221767989

Table S5 PERMANOVA adonis test results for differences in community composition between enrichments.

Comparison	Sums of Squares	Mean Squares	F Model	R2	P-value
EB2_Mackinac vs LS1_Calumet	4.6551	4.6551	79.975	0.79203	0.001
EB2_Mackinac vs LS2_Calumet	4.2407	4.2407	102.33	0.85041	0.001
LS1_Calumet vs LS2_Calumet	0.30634	0.306335	4.4685	0.1904	0.002

Table S6 PERMANOVA adonis test results for differences in community composition between media types in each enrichment. DCPET = Deconstructed polyethylene terephthalate, TPA = terephthalate, ARW = autoclaved river water, AMW = autoclaved river water, BH = Bushnell Haas.

Comparison	Sums of Squares	Mean Squares	F Model	R2	P-value
EB2_Mackinac ARW_DCPET vs AMW_DCPET	0.041557	0.041557	1.3735	0.25561	0.3
EB2_Mackinac ARW_DCPET vs BH_DCPET	0.067225	0.067225	5.1212	0.56146	0.1
EB2_Mackinac ARW_DCPET vs BH_TPA	0.062658	0.062658	2.661	0.47006	0.1

EB2_Mackinac AMW_DCPET vs BH_DCPET	0.072405	0.072405	4.0797	0.50493	0.1
EB2_Mackinac AMW_DCPET vs BH_TPA	0.066690	0.066690	2.2449	0.42801	0.2
EB2_Mackinac BH_DCPET vs BH_TPA	0.073960	0.073960	10.768	0.7821	0.1
LS1_Calumet ARW_DCPET vs AMW_DCPET	0.059252	0.059252	0.92601	0.18798	0.6
LS1_Calumet ARW_DCPET vs BH_DCPET	0.054595	0.054595	1.7155	0.30015	0.2
LS1_Calumet ARW_DCPET vs BH_TPA	0.17892	0.178920	2.4298	0.37789	0.1
LS1_Calumet AMW_DCPET vs BH_DCPET	0.059958	0.059958	1.4333	0.26381	0.2
LS1_Calumet AMW_DCPET vs BH_TPA	0.21339	0.213390	2.5512	0.38943	0.2
LS1_Calumet BH_DCPET vs BH_TPA	0.28912	0.28912	5.6161	0.58403	0.1
LS2_Calumet ARW_DCPET vs AMW_DCPET	0.053926	0.053926	5.416	0.57519	0.1
LS2_Calumet ARW_DCPET vs BH_DCPET	0.119943	0.119943	8.8885	0.74766	0.1
LS2_Calumet ARW_DCPET vs BH_TPA	0.051021	0.051021	3.7167	0.65015	0.25
LS2_Calumet AMW_DCPET vs BH_DCPET	0.30827	0.308274	36.411	0.92388	0.1
LS2_Calumet AMW_DCPET vs BH_TPA	0.105176	0.105176	17.002	0.89475	0.25
LS2_Calumet BH_DCPET vs BH_TPA	0.050781	0.050781	3.898	0.79583	0.3333

Table S7 Specific growth rates, generation times, and number of generations for each enrichment, media, and carbon type. DCPET = Deconstructed polyethylene terephthalate, TPA = terephthalate, ARW = autoclaved river water, AMW = autoclaved river water, BH = Bushnell Haas.

Group	Mean No Generations ± Std Dev	Mean Generation Time ± Std Dev	Mean Specific Growth Rate ± Std Dev

Blank DCPET AMW	0.5±0.5	Inf±NaN	0.002±0.002
Blank DCPET ARW	0.9±0.9	-135.3±331.9	0.003±0.003
Blank DCPET BH	0.8±0.3	138.5±57.2	0.002±0.001
Blank TPA BH	2.2±0.0	42.3±0.0	0.007±0.000
EB2 Mackinac DCPET AMW	5.9±1.3	16.3±3.3	0.019±0.004
EB2 Mackinac DCPET ARW	7.8±1.6	12.4±2.8	0.025±0.005
EB2 Mackinac DCPET BH	8.1±0.9	11.6±1.3	0.026±0.003
EB2 Mackinac TPA BH	6.5±0.0	14.3±0.0	0.021±0.000
LS1 Calumet DCPET AMW	5.4±0.4	17.2±1.4	0.018±0.001
LS1 Calumet DCPET ARW	5.2±1.5	19.6±6.8	0.017±0.005
LS1 Calumet DCPET BH	5.4±0.3	17.1±0.8	0.018±0.001
LS1 Calumet TPA BH	5.4±0.0	17.2±0.0	0.017±0.000
LS2 Calumet DCPET AMW	5.2±0.2	18.0±0.8	0.017±0.001
LS2 Calumet DCPET ARW	5.2±0.5	18.0±1.8	0.017±0.002
LS2 Calumet DCPET BH	5.2±0.3	17.8±0.9	0.017±0.001
LS2 Calumet TPA BH	4.9±0.0	19.0±0.0	0.016±0.000

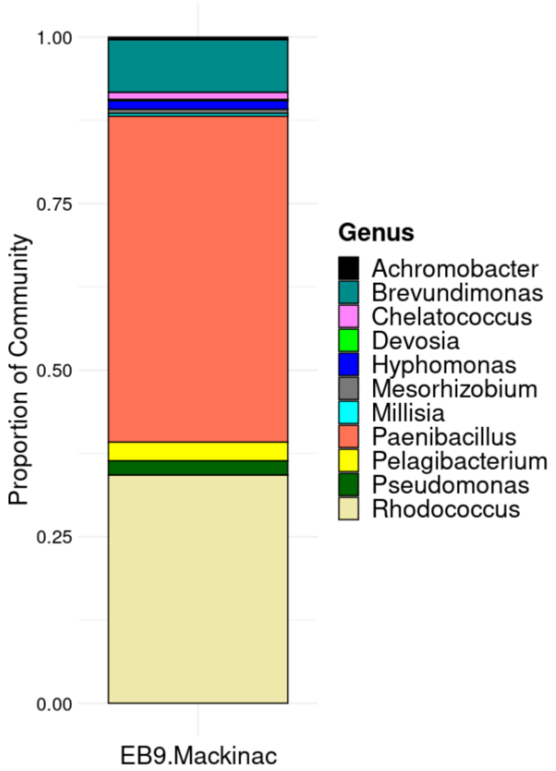

Figure S10: 16S rRNA community composition of the EB9.Mackinac community. The relative abundances of the dominant taxa are: 50.0% Paenibacillus, 34.2% Rhodococcus, 7.9% Brevundimonas, and <5% of all other members of the community.

Table S8. Format of polymers prior to deconstruction and reaction conditions for solubilization in ammonium hydroxide. All polymers were deconstructed in custom horizontal tubular reactors, except for PUF Parr, which was deconstructed in a 600 mL stirred Parr reactor. PET = polyethylene terephthalate, PUF = polyurethane foam

Format	Polymer	Reaction Conditions: Temperature (°C)	Time (min)	NH_4OH Concentration (wt %)	Solids Loading (g polymer/ mL NH_4OH)	Stirring (rpm)
Particles (5 mm)	PET	260	10	10	0.25	-
Foam Pieces	PUF Batch	200	30	16	0.0625	-
	PUF Parr	200	30	16	0.0625	150
Fabric	100% Nylon	240	60	10	0.25	-
	92/8% Nylon/Spandex	240	60	10	0.25	-
	95/5% Polyester/Spandex	240	60	10	0.25	-
	100% Polyester	240	60	10	0.25	-
Plastic Shards	Polycarbonate	240	10	10	0.25	-
Multilayer	Mylar	260	10	10	0.075	-
	Mylar (Polyolefin Extracted)	260	30	10	0.075	-

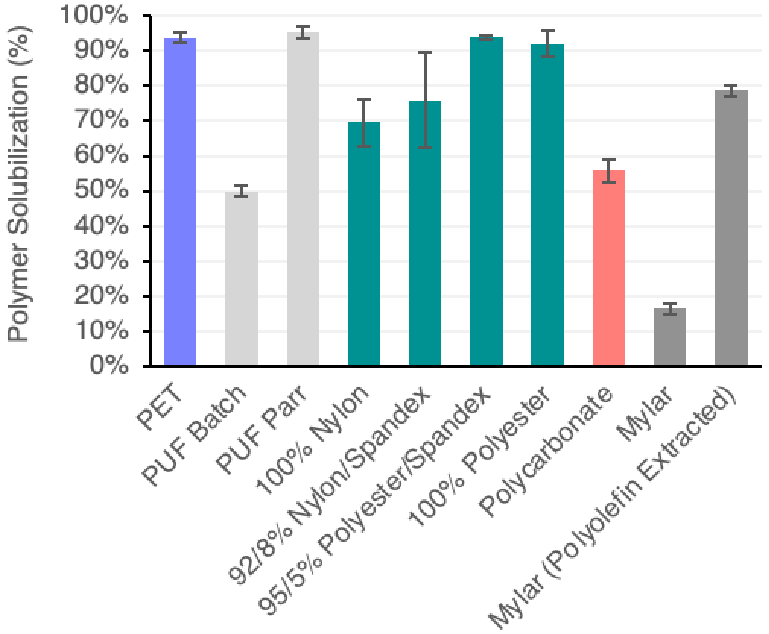

***Figure S11. Polymers were solubilized to varying degrees using ammonium hydroxide.** Reaction conditions differ for each material and are listed in SI Table 1. All solubilized material was theoretically available to the microorganisms for use as a nutrient source. Replication varied from n = 3 or n = 4 for all samples. Polymers were in various formats when deconstructed: PET = particles (<5 mm); PUF = moderate sized pieces; Fabrics (nylon/spandex and polyester spandex blends) = single squares; Polycarbonate = shards/fragments of various sizes; Mylar = small squares. PET = polyethylene terephthalate; PUF = polyurethane foam; Batch = horizontal tubular reactor; Parr = 600 mL Parr*

Chemical Processing Methods for Additional Materials

Fabrics (polyester/nylon/spandex): All fabrics were purchased from Amazon (100% polyester: 0772360, Dozier Textiles; 95% polyester/5% spandex: 0695194, E.Z. Fabric, Inc.; 100% nylon: 0725219, Vogue Group Inc.; 92% nylon/8% spandex: 0740484, 1710 S HOOPER AVE. All fabrics were manually cut into squares and loaded separately into a custom horizontal batch reactor (13.5 mL). Next, ammonium hydroxide (10 wt%) was added to the reaction vessel to achieve a solids loading of 0.25 g fabric/mL ammonium hydroxide. The reactor was heated to 240° C, and held at that temperature for a residence time of 60 min. The final product was vacuum-filtered through Whatman #42 filter paper (diameter 55 mm, pore size 2.5 µm). The solids were retained and dried at 55 °C to determine the amount solubilized. The liquid was neutralized using phosphoric acid (H_3PO_4) to reduce the pH from 10.4 to 7 and sterile filtered using a 0.2 µm PES filter before being added to the microbial culture (**Figure S9**).

Polycarbonate (PC): Recycled polycarbonate shreds were sourced from McDunnough Plastics and used without further size reduction. Polycarbonate particles were processed in a custom horizontal batch reactor (580 mL capacity) using 10 wt% NH_4OH, at 0.25 g PET/mL NH_4OH solids loading, at 240 °C for 10 minute residence time following heatup. Next, the prepared solution was vacuum-filtered through Whatman #42 filter paper (diameter 55 mm, pore size 2.5 µm). The solids were dried at 55 °C and used to determine solubilization (59.2%). The liquid was then pH-adjusted from 12.4 to 7 using phosphoric acid. The final liquid product was filtered through a 0.2 µm PES filter before being added to the microbial culture.

Flexible polyurethane foam (PUF_Batch): High density memory foam (Foamma F-6-2472H) was purchased from Amazon. The foam was passed through a paper shredder to obtain particles ranging from ~8-10 mm. Pieces of foam were loaded into a custom horizontal batch reactor, after which ammonium hydroxide (16 wt%) was added to the reactor, keeping the solids loading at 0.0625 g PUF/mL ammonium hydroxide. The reaction temperature was raised to 200 ^oC, and held at that temperature for a residence time of 30 minutes. The final product was vacuum-filtered through Whatman #42 filter paper (diameter 55 mm, pore size 2.5 µm). The solids were retained and dried at 55 °C to determine the amount solubilized (50.04%). The liquid was neutralized using phosphoric acid (H_3PO_4) to reduce the pH from 9.2 to 7 before feeding it to the microbes.

Flexible polyurethane foam (PUF Parr): Polyurethane foam was size reduced as described in the previous section and added to a 600 mL stainless steel reactor (Parr 4563) equipped with a U-shaped stirrer. Next, 16% (w/w) ammonium hydroxide was added, keeping the solids loading at 0.0625 g PUF/mL ammonium hydroxide. The reactor was heated to 200 ^oC, with stirring at 150 rpm, and a pressure of 200 psi, and held at the desired temperature for a residence time of 30 minutes. The final product was vacuum-filtered through Whatman #42 filter paper (diameter 55 mm, pore size 2.5 µm). The solids were retained and dried at 55 °C to determine the amount solubilized (95.24%). The liquid was neutralized using phosphoric acid (H_3PO_4) to reduce the pH from 9.8 to 7 before feeding it to the microbes.

Mylar Food Storage Bags: Mylar bags (Outus), consisting of three layers of PET, aluminum, and polyethylene, were purchased from Amazon. The materials were manually cut in square-shaped pieces around 10 mm in length. One set of samples (Mylar) was added to a custom horizontal batch reactor with 10 wt% ammonium hydroxide at a solid loading of 0.075 g sample/mL ammonium hydroxide. The reactor was heated to 260° C and then held for a residence time of 10 min. Next, the prepared solution was vacuum-filtered through Whatman #42 filter paper (diameter 55 mm, pore size 2.5 μm). The solids were dried at 55 °C and used to determine solubilization (17.5%). The liquid was then pH-adjusted from 10.4 to 7 using phosphoric acid. The final liquid product was filtered through a 0.2 μm PES filter before being added to the microbial culture.

An additional set of mylar bag samples (Mylar_Extracted) were processed to remove the polyethylene layer before removing the PET layer. The squares were loaded in a vial with a proprietary solvent (10:1 solvent:squares mass ratio). The vial was sealed, and the mixture was heated in a heating mantle at 150° C for 30 minutes. The squares were removed from the vial, washed with 2-propanol to remove excess solvent and polyethylene. The extracted bag squares were processed in the same manner as the squares in the previous paragraph, resulting in a solubilization of 80.3%.

Solvent Decontamination and Dehydration Methods, Idaho National Laboratory

The treatment of municipal solid waste (MSW) is a key challenge to enabling downstream conversion of valuable materials currently with the waste stream. The washing/disinfectant process is essential for creating contaminant-free feed streams for breakdown and conversion processes. However, the conventional technology, e.g. aqueous based washing and thermal drying process requires the input of water and potential detergent, and generates large volumes of aqueous waste as well as substantial energy requirement.[1, 2] A solvent-based treatment in which the solvent can be distilled for reuse has the potential to minimize energy consumption and prevent the generation of additional liquid waste thus reducing the environmental impact.[3, 4] There has been recent interest in the use of liquefied dimethyl ether (DME) as a green solvent for organic extraction, drying/dehydration, disinfection, and decontamination in general for various materials.[4-8] MSW commonly contains water and DME soluble compounds (e.g. oils and fats) that a DME based treatment step can remove and isolate. Our experimental data and previously published literature demonstrate that water can be selectively removed from brine solutions and even remove water from dry cardboard reducing the water content from 5% to 4% at ambient temperatures.[3, 9-11] DME solids treatment process, **Figure S10**, has the potential to be economically efficient and environmentally friendly by virtue of the unique physical and chemical properties of liquified DME.[6, 12]

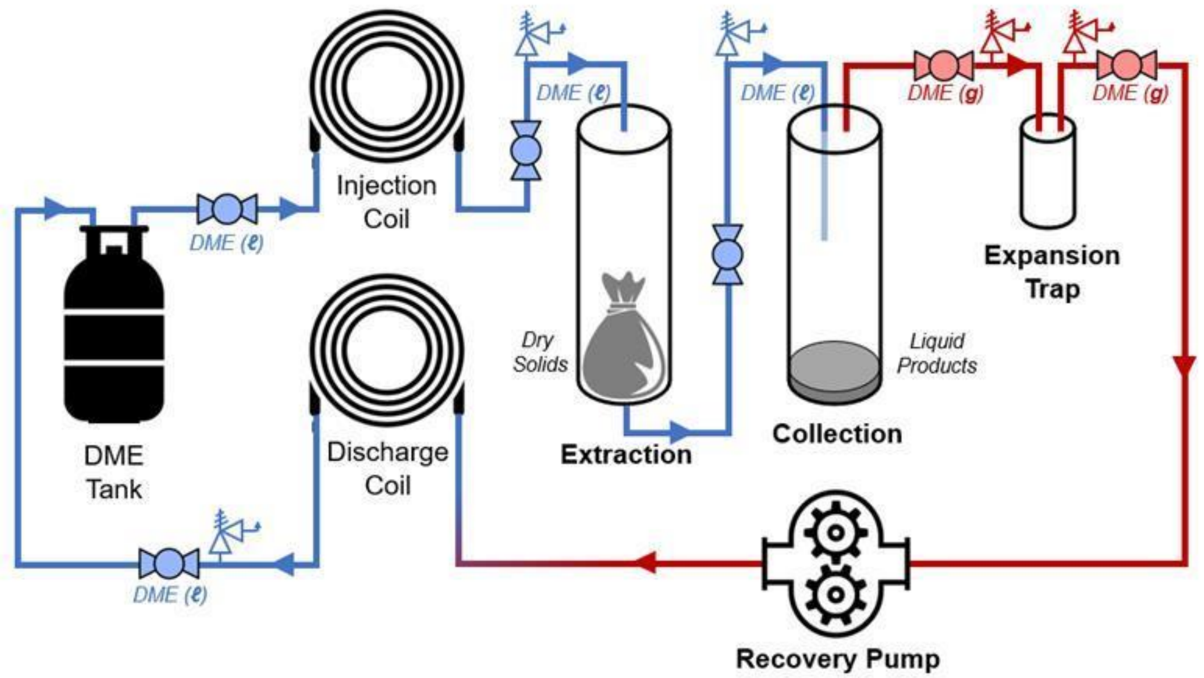

Figure S12. Schematic representation of DME solids treatment system.

DME solids treatment process comes with its challenges and requirements. The high volatility of DME facilitates solvent recovery[10]; however, the use of DME requires that washing be carried out at elevated pressures or reduced temperatures to maintain DME as a liquid. In this study, the washes were conducted at ~70 psi at 20 °C. DME is a relatively safe cleaning solvent compared to other candidates for polar extractions such as siloxanes, hydrochlorofluorocarbons (HCFCs), and bromofluorocarbons (BFCs)[13, 14]. While siloxanes have several attractive properties, they are toxic and bioaccumulate in human tissue[15]. HCFCs and BFCs aggressively attack ozone and could be harmful to the environment if released. Due to their effect on ozone, many HCFCs and BFCs have been phased out of production and are generally no longer commercially available[13, 16, 17]. In contrast, pressurized DME and many supercritical (SC) solvents like SC-CO_2 are both readily available and non-toxic. DME has the additional benefit that it is liquified with a modest increase in pressure (~70 psi), while CO_2 is only supercritical above 1,060 psi at 30 °C. Due to these higher pressure and temperature requirement, supercritical solvents are expected to require greater equipment costs and energy consumption than a corresponding DME process.

While DME is flammable, its use benefits from the well-established standards for managing flammable compressible gases such as propane.[18] Although peroxides are a concern with many ethers listed in a "Class B: Hazard Due to Peroxide Concentration" chemical, peroxide formation has not been observed under process conditions equivalent to those employed in this work. This is likely due to gas storage and handling protocols, as well as the vapor pressure of DME at operational conditions, which preclude the formation of peroxides by combining oxygen

and DME. Gaseous DME has an atmospheric lifetime of approximately 5 days and does not contribute to ozone depletion[19, 20]. Thus, DME is considered a relatively safe and environmentally benign solvent.

The US Environmental Protection Agency (EPA) does not regulate the level of DME in drinking water and the World Health Organization (WHO) does not offer specific recommendations on DME. Regulations on DME include a 2005 EPA rule issued to eliminate the need to establish a maximum permissible level for residues of DME[21]. Reported no-observed-adverse-effect concentration (NOAEC) values for ingesting DME are between 500-900 mg/kg body weight per day and are classified as *generally recognized as safe* (GRAS).[22] This is also consistent with Question No EFSA-Q-2007-186; the European Food Safety Authority (EFSA) has listed DME as a non-toxic solvent for use in the processing of protein and meat for human consumption[23].

Due to this combination of the relative ease of handling, environmental compatibility, and limited toxicity, DME is used in many commercial applications and several ubiquitous consumer products, including over-the-counter cryogenic wart remover. As such, DME is a strong candidate for large-scale industrial processes including recycling of MSW.

During the DME treatment process, solids added to the system interact with liquid DME, resulting in the transfer of mobile organics and water from the solids into the liquid DME. The mixture of solvent and extracted material is then transferred to a solvent separation compartment, where heat and reduced pressure is used to evaporate and recover DME, isolating the extracted material. The products of this process include dry solid materials free from oil and liquids and solid-free liquids which can be further separated. It is common for the liquid fraction to phase separate into two or more layers, including an aqueous layer and organic layer(s). All extracted products (liquids and solids) are disinfected by their exposure to DME.[24-26]

<u>Feedstock preparation</u>

Paper and plastic materials used in this experiment were consumer products purchased through retail outlets. Polyethylene terephthalate (PET) and polypropylene (PP) drinking cups were purchased in bulk (Dart TP16D and Karat C1011) through Amazon. Cardboard boxes and a 100’ roll of 10 mil LDPE plastic film were also purchased through Amazon. The HDPE used in this experiment came from white 5-gallon bucket lids (Price Container, Ogden, UT, item number 801165).

Materials were initially size reduced in a rotary shear (Crumbler®,Forest Concepts, Auburn, WA) to 30 mm, then further size reduced to 3-5 mm using the Crumbler® or to 450-850 µm using a knife mill (Thomas-Wiley laboratory mill Model 4, Swedesboro, NJ). All samples were shredded before DME treatment.

<u>Liquid dimethyl Ether treatment of solids</u>

A laboratory-scale hydrocarbon extraction platform (MiniMeP, ExtractionTek Solutions, Wheat Ridge, CO) was adapted for use with DME as the working solvent. To prevent seal and gasket failures the system was fitted withethylene propylene rubber (EPR) pressure relief valves and Teflon gaskets. The DME within this system operates in a closed loop, with liquified DME solvent returned to the operating tank after extraction and decontamination **Figure S10**.

All the extraction chambers (5 L sample volume) and collection vessels are constructed of insulated stainless steel with 14.605 cm inner diameter. The expansion chamber which protects the recovery pump from fluid (bump trap) has around 6.985 cm inner diameter with insulated stainless steel. The recovery pump (Haskel B3354), pneumatically driven, is capable of supplying a 150 psi pressure differential. All components were connected with Swagelok pipes and components (connections, valves, and pressure relief). Each pressure vessel featured independent pressure relief set to 450 psi. The heating unit recirculated fluid to a liquid jacket at 35 °C in the collection chamber. The cooling unit recirculated cooled fluid at -4 °C through heat exchangers labeled injection coil and discharge coil.

An example experiment involved adding 486 grams of cardboard sourced from MSW was added to the DME treatment extractions chamber. Vacuum from the recovery pump was applied to the chamber until it reached less than -10 psig. This step prevents air from mixing with the DME and having oxygen recompressed with the DME. The extraction chamber was isolated from the recovery pump and opened to the DME storage tank, and 2 kg was transferred from the DME storage tank to the extraction chamber. The liquid DME completely submerged the solids and steeped for 20 min. The liquid DME, now containing water and oil, was vacuum transferred from the extraction chamber to the collection vessel where heat was supplied for DME separation/evaporation. The compressed gas is directed to a discharge coil that condenses and cools the DME for storage in the DME tank. Around 294 grams of solid sample were recovered from the extraction chamber. Around 200 mL of liquids (DME soluble and extracted) were recovered from the collection chamber.

Idaho National Laboratory Method References:

1. Brunetti, L.; Giametta, F.; Catalano, P.; Villani, F.; Fioralba, J.; Fucci, F.; La Fianza, G., Energy consumption and analysis of industrial drying plants for fresh pasta process. *Journal of Agricultural Engineering* **2015,** *46* (4), 167-171.
2. Dettenkofer, M.; Spencer, R. C., Importance of environmental decontamination – a critical view. *Journal of Hospital Infection* **2007,** *65*, 55-57.
3. Bauer, M. C.; Kruse, A., The use of dimethyl ether as an organic extraction solvent for biomass applications in future biorefineries: A user-oriented review. *Fuel* **2019,** *254*, 115703.
4. Kanda, H.; Li, P.; Ikehara, T.; Yasumoto-Hirose, M., Lipids extracted from several species of natural blue–green microalgae by dimethyl ether: Extraction yield and properties. *Fuel* **2012,** *95*, 88-92.

5. Levine, I., Method of simultaneously defatting, dehydrating, and eliminating bacteria from foodstuffs. Google Patents: 1974.

6. Li, P.; Makino, H., Liquefied dimethyl ether: An energy-saving, green extraction solvent. In *Alternative Solvents for Natural Products Extraction*, Springer: 2014; pp 91-106.

7. Kanda, H.; Makino, H., Energy-efficient coal dewatering using liquefied dimethyl ether. *Fuel* **2010,** *89* (8), 2104-2109.

8. Kanda, H.; Ando, D.; Hoshino, R.; Yamamoto, T.; Wahyudiono; Suzuki, S.; Shinohara, S.; Goto, M., Surfactant-free decellularization of porcine aortic tissue by subcritical dimethyl ether. *ACS omega* **2021,** *6* (20), 13417-13425.

9. McNally, J. S.; Foo, Z. H.; Deshmukh, A.; Orme, C. J.; Lienhard, J. H.; Wilson, A. D., Solute displacement in the aqueous phase of water–NaCl–organic ternary mixtures relevant to solvent-driven water treatment. *RSC advances* **2020,** *10* (49), 29516-29527.

10. Deshmukh, A.; Foo, Z. H.; Stetson, C.; Lee, H.; Orme, C. J.; Wilson, A. D.; Lienhard, J. H., Thermodynamics of solvent-driven water extraction from hypersaline brines using dimethyl ether. *Chemical Engineering Journal* **2022,** *434*, 134391.

11. Stetson, C.; Prodius, D.; Lee, H.; Orme, C.; White, B.; Rollins, H.; Ginosar, D.; Nlebedim, I. C.; Wilson, A. D., Solvent-driven fractional crystallization for atom-efficient separation of metal salts from permanent magnet leachates. *Nature Communications* **2022,** *13* (1), 1-9.

12. Levine, I. Method of simultaneously defatting,dehydrating,and eliminating bacteria from foodstuffs. 1973-03-02, 1973.

13. Durkee, J. B., Cleaning with solvents. In *Developments in Surface Contamination and Cleaning*, Elsevier: 2008; pp 759-871.

14. Durkee, J. B.; Williams, L. L., On The Value of CO2 in Cleaning Operations.

15. Mojsiewicz-Pieńkowska, K.; Krenczkowska, D., Evolution of consciousness of exposure to siloxanes—review of publications. *Chemosphere* **2018,** *191*, 204-217.

16. Hegglin, M. I.; Fahey, D. W.; McFarland, M.; Montzka, S. A.; Nash, E. R., *Twenty Questions and Answers About the Ozone Layer: 2014 Update-Scientific Assessment of Ozone Depletion: 2014*. 2015.

17. Protocol, M., Montreal protocol on substances that deplete the ozone layer. *Washington, DC: US Government Printing Office* **1987,** *26*, 128-136.

18. Hoshino, R.; Wahyudiono, Simultaneous Extraction of Water and Essential Oils from Citrus Leaves and Peels Using Liquefied Dimethyl Ether. *Journal of Nutrition & Food Sciences* **2014,** *4* (5).

19. Oshita, K.; Takaoka, M.; Nakajima, Y.; Morisawa, S.; Kanda, H.; Makino, H.; Takeda, N., Characteristics of biosolids in dimethyl ether dewatering method. *Water Environ Res* **2012,** *84* (2), 120-7.

20. Kanda, H.; Morita, M.; Makino, H.; Takegami, K.; Yoshikoshi, A.; Oshita, K.; Takaoka, M.; Morisawa, S.; Takeda, N., Deodorization and dewatering of biosolids by using dimethyl ether. *Water Environ Res* **2011,** *83* (1), 23-5.

21. Dimethyl Ether; Exemption from the Requirement of a Tolerance. In *40 CFR 80*, Agency, E. P., Ed. Federal Register: Federal Register, 2005; Vol. 70 FR 28436.

22. Tallon, S. *GRAS Notice for the Use of Dimethyl Ether as an Extraction Solvent*; Food and Drug Administration: Office of Food Additive Safety, 2017.

23. Arturo Anadón, D. B., Mona-Lise Binderup, Wilfried Bursch, Laurence Castle, Riccardo Crebelli, Karl-Heinz Engel, Roland Franz, Nathalie Gontard, Thomas Haertlé, Trine Husøy, Klaus-Dieter Jany, Catherine Leclercq, Jean-Claude Lhuguenot, Wim Mennes, Maria Rosaria Milana, Karla Pfaff, Kettil Svensson, Fidel Toldrá, Rosemary Waring, Detlef Wölfle, Safety in use of dimethyl ether as an extraction solvent. *The EFSA Journal* **2009,** *984*, 1-13.

24. Levine, I. E. Methoxymethane sterilization method. US4048343A, 1977/09/13/, 1977.

25. Levine, I. E. Methoxymethane sterilization method. US3900288A, 1975/08/19/, 1975.

26. Levine, I. Method of simultaneously defatting,dehydrating,and eliminating bacteria from foodstuffs. US3795750A, 1974/03/05/, 1974.
